# Efficient Somatic Cell Nuclear Transfer by Overcoming Both Pre‐ and Post‐Implantation Epigenetic Barriers

**DOI:** 10.1002/advs.202504669

**Published:** 2025-07-08

**Authors:** Yamei Li, Shiyu Sun, Yuting Xu, Jixiang Zhang, Yi Du, Yaxin Cao, Zhaodi Liao, Yali Xie, Xinyan Bian, Jiantao Huang, Meijiao Wang, Zhen Liu, Qiang Sun, Falong Lu

**Affiliations:** ^1^ State Key Laboratory of Molecular Developmental Biology Institute of Genetics and Developmental Biology Chinese Academy of Sciences Beijing 100101 China; ^2^ University of Chinese Academy of Sciences Beijing 100049 China; ^3^ Institute of Neuroscience CAS Key Laboratory of Primate Neurobiology State Key Laboratory of Neuroscience Center for Excellence in Brain Science and Intelligence Technology Chinese Academy of Sciences Shanghai 200031 China; ^4^ Shanghai Center for Brain Science and Brain‐Inspired Intelligence Technology Shanghai 201210 China

**Keywords:** embryo, epigenetic barrier, imprinting, inner cell mass, SCNT, tetraploid complementation, trophectoderm

## Abstract

Mammalian cloning through somatic cell nuclear transfer (SCNT) involves reprogramming terminally differentiated cells into totipotent embryos. Epigenetic barriers inherited from somatic cells impede reprogramming efficiency and lead to low SCNT embryo development rates. Recent studies have identified two primary types of epigenetic barriers in mouse SCNT embryos, defective pre‐implantation development due to abnormal gene expression around zygotic genome activation, associated with aberrant H3K9me3, H3K4me3, and histone acetylation, and defective post‐implantation development due to loss of H3K27me3‐mediated non‐canonical imprinting. Despite these findings, effective strategies to overcome these barriers in a single embryo have not been established. Here, Kdm4d and Kdm5b overexpression are combined with TSA treatment to overcome epigenetic barriers in pre‐implantation development, while using tetraploid complementation to replace extraembryonic lineage cells, thereby overcoming imprinting defects critical for post‐implantation development. This approach resulted in ≈30% full‐term development efficiency of reconstructed embryos. The strategy not only represents the highest SCNT efficiency achieved in mammals but also enhances the feasibility of efficient mammal cloning.

## Graphical abstract

Mammalian cloning via SCNT is limited by several epigenetic barriers. Kdm4d/Kdm5b overexpression plus TSA treatment overcomes epigenetic barriers during pre‐implantation development, while tetraploid complementation replaces defective extraembryonic lineages to bypass H3K27me3 imprinting loss to overcome epigenetic barriers in post‐implantation development. This combined strategy yields ≈30% full‐term development of mouse reconstructed SCNT embryos, greatly advancing mammalian cloning.

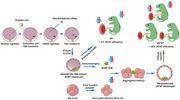

## 1 Introduction

Somatic cell nuclear transfer (SCNT), the process of transferring a somatic nucleus into an enucleated oocyte, reprograms terminally differentiated somatic cells into totipotent embryos.^[^ ^1^, ^2^, ^3^, ^4^, ^5^, ^6^ ^]^ SCNT‐mediated mammalian cloning holds tremendous potential for agro‐biotechnology, endangered species conservation, disease model production, and regenerative medicine.^[^ ^7^, ^8^, ^9^, ^10^, ^11^ ^]^ More than 20 mammalian species have been successfully cloned through SCNT,^[^ ^3^, ^7^ ^]^ including the cloning of non‐human primates.^[^ ^12^, ^13^, ^14^ ^]^ However, the application of SCNT remains highly limited due to the low efficiency of full‐term development in cloned embryos.^[^ ^7^ ^]^ For example, the full‐term developmental efficiency of mouse SCNT embryos is ≈1% using the conventional SCNT technique.^[^ ^15^, ^16^ ^]^

Cloned embryos share the same DNA sequence as naturally fertilized embryos. Therefore, the developmental defects in cloned embryos are most likely due to epigenetic aberrations. Studies have shown that aberrant H3K9me3, H3K4me3, histone acetylation, and chromatin organization are major epigenetic barriers for proper zygotic genome activation and pre‐implantation development of SCNT embryos.^[^ ^11^, ^16^, ^17^, ^18^, ^19^, ^20^ ^]^ Treatment of the SCNT embryos with trichostatin A (TSA),^[^ ^20^, ^21^, ^22^ ^]^ a histone deacetylase inhibitor, removal of H3K9me3 through overexpression of Kdm4d or Kdm4b,^[^ ^11^, ^16^, ^17^ ^]^ or removal of H3K4me3 by Kdm5b overexpression^[^ ^17^ ^]^ in SCNT embryos significantly improves pre‐implantation development and increase the mouse cloning efficiency up to 8%. Importantly, Kdm4d treatment,^[^ ^16^ ^]^ combined treatment with Kdm4d and TSA,^[^ ^16^ ^]^ or combined treatment with Kdm4b and Kdm5b,^[^ ^17^ ^]^ yield blastocyst rates comparable to those of naturally fertilized embryos (NF embryos). Moreover, Kdm4 treatment is also effective in improving pre‐implantation development of SCNT embryos in non‐human primates, bovines, and pigs.^[^ ^13^, ^23^, ^24^ ^]^

In addition to epigenetic barriers impeding pre‐implantation development, SCNT embryos exhibit epigenetic defects that impede post‐implantation development, including abnormal X chromosome inactivation^[^ ^25^, ^26^ ^]^ and loss of H3K27me3‐mediated non‐canonical imprinting.^[^ ^25^, ^27^, ^28^, ^29^, ^30^ ^]^ In normal pre‐implantation embryos and placentas, the paternal X chromosome is selectively inactivated while the maternal X chromosome remains active, a process known as imprinted X chromosome inactivation.^[^ ^31^, ^32^ ^]^ However, in female SCNT embryos, both X chromosomes undergo inactivation, and in male embryos, the single X chromosome is inactivated due to ectopic expression of *Xist*.^[^ ^26^ ^]^ These defects are caused by loss of H3K27me3‐mediated non‐canonical imprinting at the *Xist* locus in SCNT embryos.^[^ ^25^, ^33^ ^]^ Restoring normal X chromosome inactivation by knocking out *Xist* from one X chromosome in donor somatic cells significantly improves cloning efficiency.^[^ ^25^, ^26^ ^]^ Combined Kdm4d treatment and Xist heterozygous knockout result in full‐term development rates of up to 23.5%. However, defects persist in post‐implantation development, including abnormally large placentas with expanded spongiotrophoblast (ST) layers and irregular boundaries between spongiotrophoblast and labyrinthine (LB) cells.^[^ ^15^, ^25^, ^29^, ^34^ ^]^ Mimicking imprinting of key H3K27me3 imprinted genes has been shown to fix these placental defects,^[^ ^27^, ^28^, ^29^ ^]^ resulting in significant improvements in SCNT efficiency up to 14% and the formation of normal placentas from somatic fibroblasts with a quadruple mono‐allelic deletion of *Sfmbt2*, *Jade1*, *Gab1*, and *Smoc1*.^[^ ^28^ ^]^

Despite these advancements, it remains challenging to overcome all major epigenetic barriers in a single SCNT embryo. In particular, mimicking H3K27me3‐mediated imprinting as described above, while effective, is extremely technically difficult and introduces genetic changes in the animals.^[^ ^27^, ^28^, ^29^ ^]^ One unique feature of H3K27me3‐mediated non‐canonical imprinting is its persistence in the placenta, while it is lost in the fetus.^[^ ^31^ ^]^ Therefore, the non‐canonical imprinting defects are in the placenta but not in the fetus. Since the placenta primarily develops from trophectoderm (TE) cells, while somatic tissues arise from the inner cell mass (ICM), we hypothesized that replacing the TE lineage of SCNT blastocysts with normal TE cells could fix the H3K27me3‐mediated non‐canonical imprinting defects. The tetraploid complementation assay is the gold standard to test the pluripotency of embryonic stem cells or induced pluripotent stem cells, in which tetraploid cells will form extraembryonic tissues while diploid pluripotent cells will form the fetus.^[^ ^35^, ^36^, ^37^, ^38^ ^]^ Replacing trophoblast cells in cloned blastocysts by tetraploid complementation has been shown to improve cloning efficiency up to 15.7%,^[^ ^39^ ^]^ though the underlying molecular mechanisms remain unclear.

In this study, we developed an easily applicable strategy to improve both pre‐implantation and post‐implantation development of SCNT embryos without requiring genetic manipulation of donor somatic cells, achieving a full‐term development rate of 30% for transferred SCNT embryos.

## 2 Results

### 2.1 TSA Treatment Combined with Injection of *Kdm4d* and *Kdm5b* mRNA Improves Pre‐Implantation Development of SCNT Embryos

**Figure 1:**
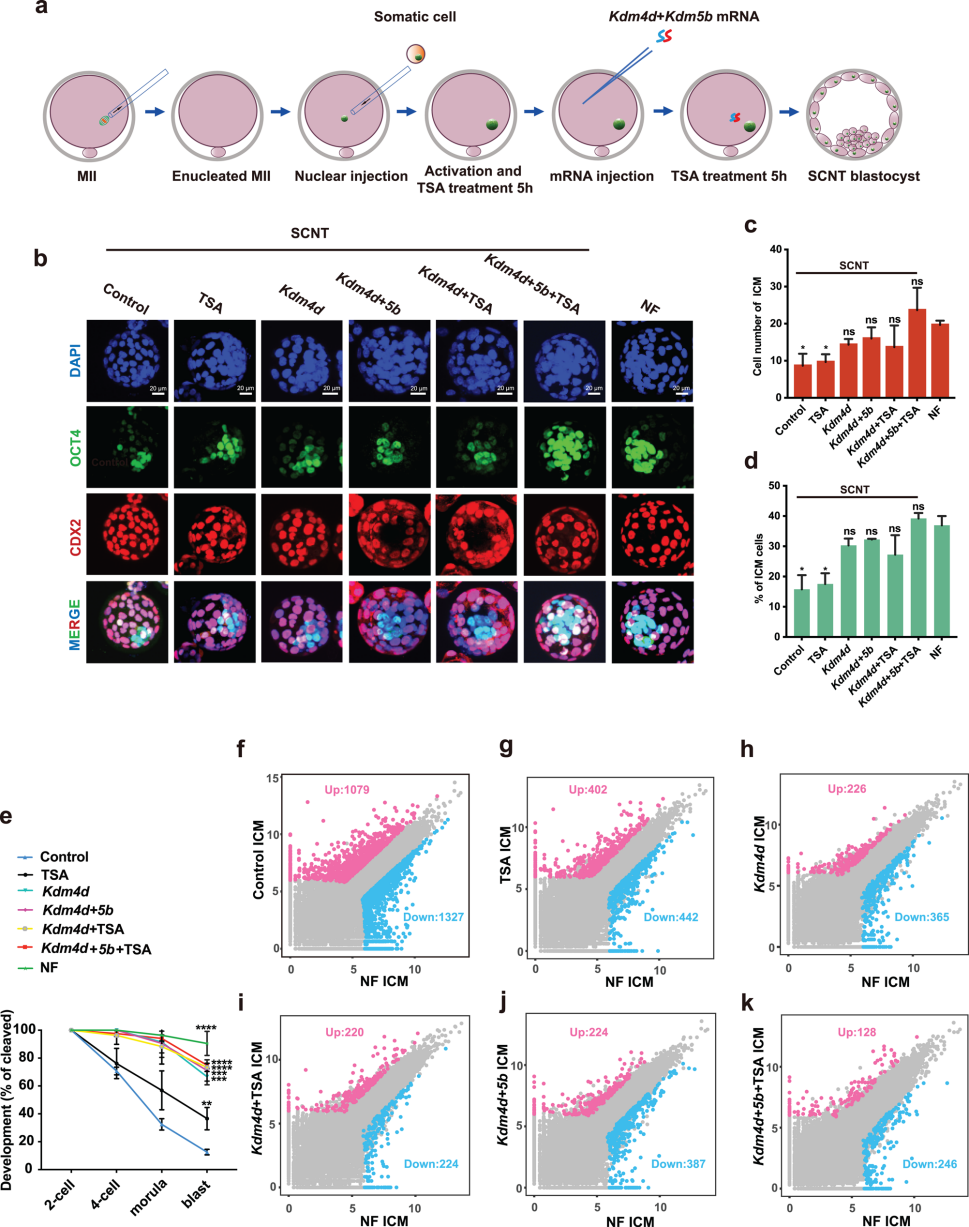
TSA treatment combined with *Kdm4d* and *Kdm5b* mRNA injection improves preimplantation development of SCNT embryos. a) Schematic illustration of cloned embryo preparation using the *Kdm4d*+*5b*+TSA method. Somatic cells are injected into enucleated MII oocytes to form reconstructed SCNT embryos. The reconstructed SCNT embryos are treated with TSA for 10 h and injected with *Kdm4d* and *Kdm5b* mRNA at the indicated time point after activation. b) Immunofluorescence of SCNT embryos (Control, TSA treated, *Kdm4d* treated, *Kdm4d*+*5b* treated, *Kdm4d*+TSA treated, and *Kdm4d*+*5b*+TSA treated) and naturally fertilized (NF) embryos. Blue indicates DNA stained by DAPI, green indicates OCT4 stained by anti‐OCT4 labeling the inner cell mass, and red indicates CDX2 stained by anti‐CDX2 labeling the trophoblasts. Merged images are shown at the bottom. The scale bar represents 20 µm. c, d) Bar graphs showing the cell number of ICM c) and the ratio of ICM cells d) in SCNT embryos (Control, TSA treated, *Kdm4d* treated, *Kdm4d*+*5b* treated, *Kdm4d*+TSA treated, and *Kdm4d*+*5b*+TSA treated) and NF embryos. Three biological replicates were analyzed for each group. Error bars represent SD. Statistical analysis was performed using Student's *t*‐test. ^*^ *p* < 0.05, “ns” represents not significant. e) Development rates at different stages for SCNT embryos (Control, TSA treated, *Kdm4d* treated, *Kdm4d*+*5b* treated, *Kdm4d*+TSA treated, and *Kdm4d*+*5b*+TSA treated) and NF embryos. Three biological replicates were analyzed for each group. Error bars represent SD. Statistical analysis of blastocyst rate was performed using Student's *t*‐test. ^**^ *p* < 0.01, ^***^ *p* < 0.001, ^****^ *p* < 0.0001. f–k) Differentially expressed genes in Control SCNT ICM versus NF ICM (f), TSA‐treated SCNT ICM versus NF ICM (g), *Kdm4d*‐treated SCNT ICM versus NF ICM (h), *Kdm4d*+TSA‐treated SCNT ICM versus NF ICM (i), *Kdm4d*+*5b* treated SCNT ICM versus NF ICM (j), and *Kdm4d*+*5b*+TSA treated SCNT ICM versus NF ICM (k). Differentially expressed genes were called by DESeq2 with fold change > 4, padj < 0.05, and basemean > 200.

Aberrant H3K9me3, H3K4me3, and histone acetylation are major barriers for pre‐implantation development in SCNT embryos.^[^ ^11^, ^16^, ^17^, ^18^, ^20^, ^21^, ^22^ ^]^ Therefore, we hypothesized that fixing these problems together could improve pre‐implantation development in SCNT embryos. We treated mouse SCNT embryos with TSA and co‐injected *Kdm4d* and *Kdm5b* mRNA (**Figure** **1**a), which we referred to as the “cocktail method” in this study. We compared these embryos with SCNT embryos without additional treatment (Control group), SCNT embryos treated with TSA only (TSA group), SCNT embryos injected with *Kdm4d* mRNA (*Kdm4d* group), SCNT embryos injected with both *Kdm4d* and *Kdm5b* mRNA (*Kdm4d+5b* group), SCNT embryos treated with TSA and *Kdm4d* mRNA (*Kdm4d*+TSA group), and naturally fertilized embryos (NF group). Cumulus cells from B6D2F1 mice were used as donor cells for SCNT. Expression of OCT4, a key transcription factor for pluripotency in the inner cell mass (ICM), is an indicator of cloning efficiency.^[^ ^22^, ^40^, ^41^, ^42^, ^43^ ^]^ There were no significant differences in the number of ICM cells between the SCNT groups and the NF group, except for fewer cells in the control and TSA‐treated SCNT blastocysts (Figure 1b–d; Table S1, Supporting Information). The blastocyst rate of cocktail‐treated embryos was 75%, which was comparable to that of the *Kdm4d* group, *Kdm4d+5b* group, and *Kdm4d*+TSA group SCNT embryos. In contrast, the blastocyst rate for the control and TSA groups was 13% and 37%, respectively (Figure 1e; Figure S1, Table S2, Supporting Information). Therefore, the injection of *Kdm4d* mRNA, with or without TSA or *Kdm5b*, improves the pre‐implantation development of SCNT embryos.

The fetus of a post‐implantation embryo is from the ICM of a blastocyst. To further characterize the SCNT embryos, we performed RNA‐seq analysis on ICMs from each of the groups of SCNT embryos, using ICMs from NF embryos as a reference (Figure S2, Supporting Information). ICMs from control SCNT embryos showed 1,079 upregulated and 1,327 downregulated genes (Figure 1f). TSA treatment reduced the number of differentially expressed genes (DEGs) (Figure 1g), while *Kdm4* treatment led to even fewer DEGs (Figure 1h). Combining TSA or *Kdm5* with *Kdm4* treatment resulted in slightly fewer DEGs (Figure 1i,j). Importantly, the cocktail method treatment that combines *Kdm4*, *Kdm5*, and TSA resulted in the fewest DEGs in SCNT ICMs compared to NF embryos (Figure 1k). Gene Ontology (GO) analyses of biological processes (GOBP) of the DEGs indicate that several biological processes are significantly dysregulated in control SCNT ICMs, while these dysregulations are alleviated by *Kdm4d* + *Kdm5b* + TSA cocktail treatment (Figure S3, Supporting Information). Together, these results suggest that the ICMs from cocktail‐treated SCNT embryos are more similar to those from NF embryos, indicating that the cocktail method is effective in improving the pre‐implantation development of SCNT embryos.

### 2.2 Reconstruction of Trophoblast‐Replaced SCNT Embryos

Our prior studies demonstrated the loss of H3K27me3‐mediated non‐canonical genomic imprinting in mouse SCNT embryos.^[^ ^25^, ^28^ ^]^ We and others have also demonstrated that mimicking H3K27me3 imprinting in donor somatic cells, through the heterozygous knockout of key imprinted genes, can reduce placental defects and significantly improve post‐implantation development and full‐term birth rates of SCNT embryos.^[^ ^27^, ^28^, ^29^ ^]^ However, mimicking H3K27me3‐mediated imprinting by quadruple heterozygous knockout is technically extremely difficult to perform and introduces multiple genetic changes in the donor cells. Therefore, we turned to trophectoderm (TE) replacement using tetraploid complementation to investigate if it could fix the H3K27me3‐mediated non‐canonical imprinting defects.

**Figure 2:**
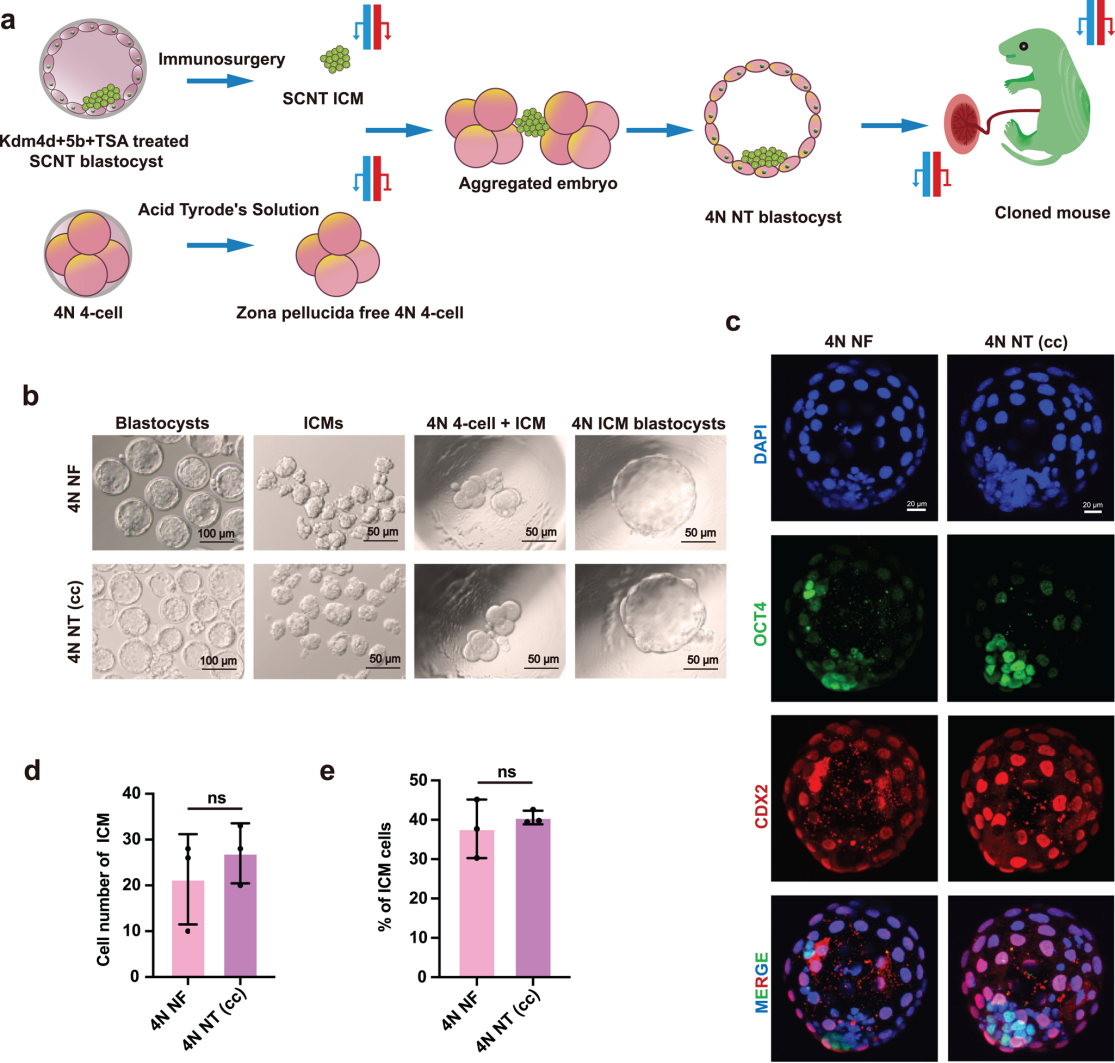
Blastocysts reconstructed from SCNT‐derived inner cell mass using tetraploid complementation exhibit normal morphology. a) Schematic illustration of tetraploid complementation for SCNT blastocyst. The inner cell mass, obtained by immunosurgery from one SCNT embryo, is aggregated with two 4‐cell tetraploid embryos without zona pellucida, resulting in reconstructed 4N NT embryos. The reconstructed embryos are transferred to the surrogate mothers until cesarean delivery at E19.5. Blue bars represent paternal alleles, and red bars represent maternal alleles, indicating the expression pattern of H3K27me3‐controlled non‐canonical imprinting genes. b) Images of reconstructed 4N NF and 4N NT (cc) embryos. Scale bars represent 100 µm or 50 µm. c) Immunofluorescence of reconstructed 4N NF and 4N NT (cc) blastocysts. Blue indicates DNA stained by DAPI, green indicates OCT4 stained by anti‐OCT4 labeling the inner cell mass, and red indicates CDX2 stained by anti‐CDX2 labeling trophoblasts. Merged images are shown at the bottom. Scale bars represent 20 µm. d, e) Bar graphs showing the number of ICM cells (d) and the ratio of ICM cells (e) in 4N NF embryos and 4N NT (cc) blastocysts. Three biological replicates were analyzed for each group. Error bars represent SD. Statistical analysis was performed using Student's *t*‐test. “ns” represents not significant.

We first confirmed the successful tetraploid complementation using ICMs from NF embryos, which resulted in normal live pups (Figure S4, Supporting Information), with a full‐term birth rate similar to previous reports (Figure S4c, Supporting Information).^[^ ^39^, ^44^, ^45^ ^]^ To test if replacing TE by tetraploid complementation could fix the H3K27me3‐mediated non‐canonical imprinting defects, we reconstructed embryos using ICM from SCNT embryos (cumulus cells as donors with TSA, *Kdm4d*, and *Kdm5b* treatment) and wild‐type tetraploid embryos (**Figure** **2**a). The ICM and TE of the reconstructed blastocysts appeared normal (Figure 2b,c). In addition, the reconstructed embryos had a normal number of ICMs, comparable to that in embryos constructed with NF ICMs and 4N embryos (Figure 2d,e; Table S3, Supporting Information). In these reconstructed embryos, we expected the fetus to come from the SCNT embryo, while the placenta would come from the WT 4N embryos. This was confirmed by fluorescent reporter tracking and fluorescence‐activated cell sorting (FACS) analysis, which showed that the 4N cells contributed to the placenta but minimally to the fetus (Figure S5, Supporting Information).

**Figure 3:**
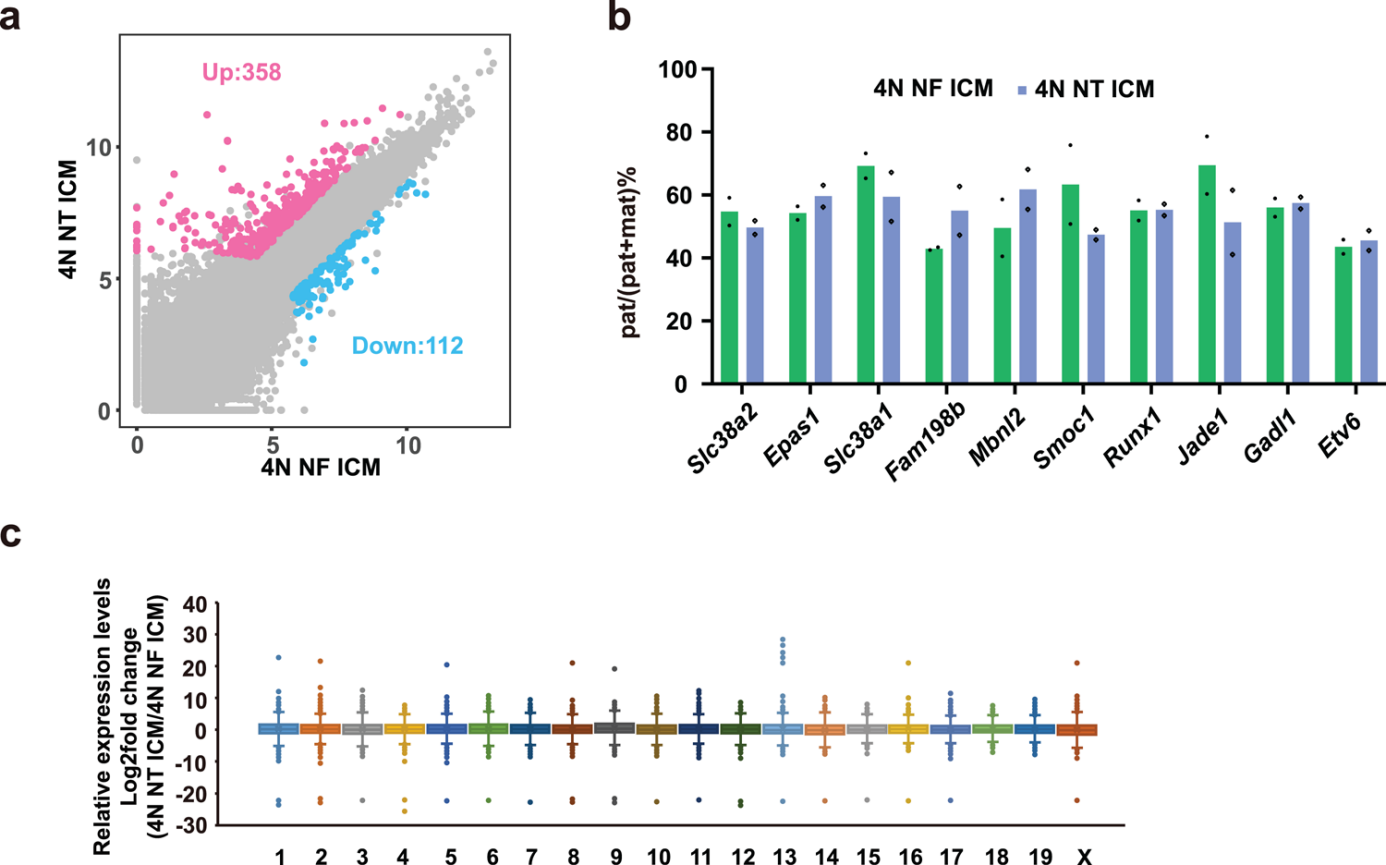
Non‐canonical imprinting is normal in the inner cell mass of 4N NT embryos. a) Differentially expressed genes in 4N NT ICM versus 4N NF ICM. Differentially expressed genes were called by DESeq2 with a fold change > 4, padj < 0.05, and basemean > 200. b) Bar graphs showing the ratio of paternal expression (number of paternal transcripts divided by the sum of paternal and maternal transcripts) for the H3K27me3‐controlled non‐canonical imprinting genes in 4N NF ICM and 4N NT ICM, as determined by SNP tracking. Dots indicate the value of two biological replicates. c) Box plot showing the relative expression of genes on individual chromosomes between 4N NT ICM and 4N NF ICM. The middle line in each box represents the median. Box edges and whiskers represent the 25th/75th and 2.5th/97.5th percentiles, respectively. The value represents the average value of two biological replicates.

To further analyze the reconstructed blastocysts, we performed RNA‐seq analysis on the ICMs from the 4N NT and 4N NF reconstructed embryos (Figure S6, Supporting Information). In the ICMs from 4N NT embryos, 358 genes were upregulated, and 112 genes were downregulated compared to the 4N NF embryos (**Figure** **3**a). Ten non‐canonical imprinted genes, whose allelic origin of mRNA could be distinguished by SNP analysis, were expressed at detectable levels. These genes exhibited a similar pattern of largely loss of imprinting in both 4N NT and 4N NF embryos (Figure 3b), consistent with previous findings of starting of loss of non‐canonical imprinting in the ICM.^[^ ^31^ ^]^ In addition, the expression levels of X chromosome genes were largely normal, as expected from the reactivation of X chromosomes in the ICMs (Figure 3c).^[^ ^46^ ^]^ These results confirm the successful reconstruction of blastocysts from SCNT ICMs and 4N embryos.

### 2.3 Replacement of Trophoblast in SCNT Embryos Fixes Imprinting Defects in TE

**Figure 4:**
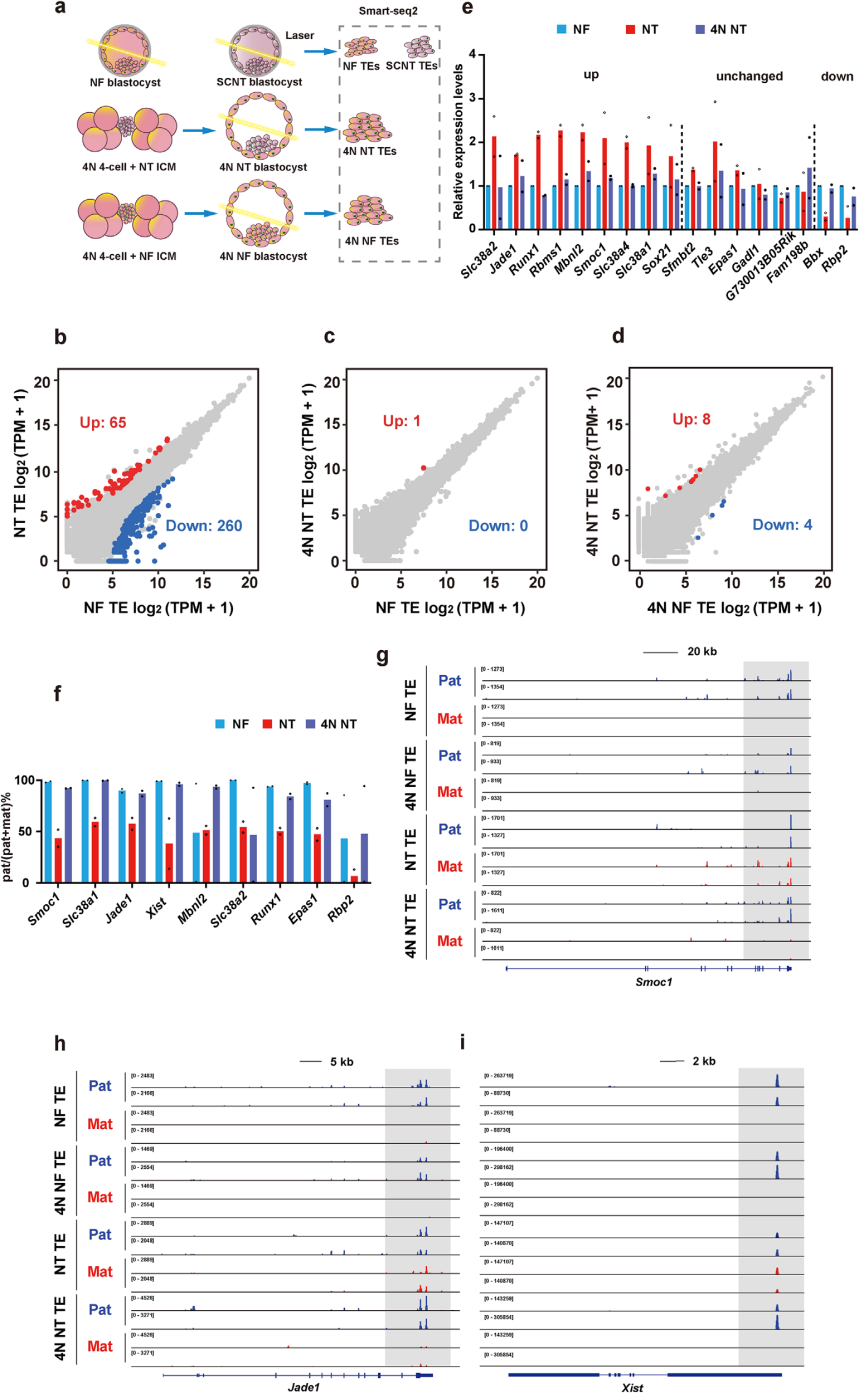
Replacement of SCNT trophoblast by tetraploid complementation corrects imprinting defects in TEs. a) Schematic illustration of the collection of TEs from NF, SCNT, 4N NT, and 4N NF blastocysts for RNA‐seq. Cumulus cells were used as donor cells for SCNT. TEs were collected by laser cutting (see methods for details). b–d) Differential gene expression in NF versus NT (b), NF versus 4N NT (c), and 4N NF versus 4N NT (d). Differentially expressed genes were called by DESeq2 with a fold change > 4 and *p* < 0.01. e) Bar graphs showing the relative gene expression levels of H3K27me3‐controlled non‐canonical imprinting genes (17 detectable genes in the TE from NF embryos, TPM > 1; genes on the X chromosome are excluded from this analysis due to the mixed male and female embryos in the RNA‐seq experiment) in NF, NT, and 4N NT TEs. The relative expression level in NF TEs for each gene is set to 1. The dashed lines separate up‐regulated, down‐regulated, and unchanged genes between SCNT and NF TEs (FC > 1.5). Dots indicate the value of two biological replicates. f) Bar graphs showing the ratio of paternal expression (number of paternal transcripts divided by the sum of paternal and maternal transcripts) for the H3K27me3‐controlled non‐canonical imprinting genes in NF, NT, and 4N NT TEs, identified by SNP tracking. Dots indicate the value of two biological replicates. g–i) Genome browser views of RNA‐seq data for H3K27me3‐controlled non‐canonical imprinting genes, including *Smoc1* (g), *Jade1* (h), and *Xist* (i). These genes are paternally expressed in NF, 4N NF, and 4N NT embryos, but biallelically expressed in NT embryos. Paternal expression is shown in blue, and maternal expression is shown in red. Scale bars are shown at the top of each view.

Since the placenta is from the 4N cells, we expected that the H3K27me3‐mediated non‐canonical imprinting would become normal in TEs of the reconstructed embryos compared to those from NT embryos. To test this hypothesis, we performed RNA‐seq on TEs from NF embryos, NT embryos (treated with the cocktail method), 4N and NT‐ICM (treated with the cocktail method) reconstructed embryos, and 4N and NF‐ICM reconstructed embryos (**Figure** **4**a; Figure S7, Supporting Information).

When comparing TE from NT embryos to that from NF embryos, there were 65 genes upregulated and 260 genes downregulated (fold change > 4, *p* < 0.01) (Figure 4b). These differentially expressed genes are likely related to the post‐implantation developmental defects seen in SCNT embryos. Interestingly, TE from 4N and NT‐ICM reconstructed embryos showed almost identical transcriptional profiles to TE from NF embryos (Figure 4c). Furthermore, TE from 4N and NT‐ICM reconstructed embryos (4N NT) was also transcriptionally nearly identical to TE from 4N and NF‐ICM reconstructed embryos (4N NF) (Figure 4d). These results suggest that our strategy can fix the transcriptional abnormalities in the TE of reconstructed SCNT embryos.

To confirm the correction of imprinting defects, we examined the transcription of imprinted genes. For the H3K27me3‐controlled non‐canonical imprinted genes detectable (TPM > 1) in the TE from NF embryos, a number of them showed differential expression in TE from NT embryos (fold change > 1.5) (Figure 4e). Importantly, these differential expressions were fixed in the TE from 4N and NT‐ICM reconstructed embryos (Figure 4e). The donor cells used for SCNT were from the BDF1 background (DBA2 as father and C57BL/6 as mother). Therefore, we could trace the parental origin of some imprinted genes using SNPs between the maternal and paternal genomes. For the SNP‐trackable non‐canonical imprinted genes, all showed paternal‐specific expression in TE from NF embryos, while biallelic expression was observed in NT embryos (Figure 4f–i; Figure S8, Supporting Information), consistent with our previous findings in NF and NT blastocysts or placentas.^[^ ^25^, ^28^ ^]^ Importantly, the loss of paternal‐specific expression of non‐canonical imprinted genes in TE from SCNT embryos was fixed in TE from 4N and NT‐ICM reconstructed embryos for all SNP trackable non‐canonical imprinted genes (Figure 4f–i; Figure S8, Supporting Information). For example, for *Smoc1*, *Jade1*, and *Xist*, nearly all SNP trackable reads were from the paternal allele in the TE from NF or 4N NF embryos, whereas they were biallelically expressed in NT embryos, confirming the loss of imprinting in NT embryos (Figure 4g–i). In the TE from 4N NT embryos, however, they were paternally expressed, confirming the fixation of their imprinted expression in the reconstructed embryos (Figure 4g–i).

Taken together, we reveal that replacing the TE of SCNT embryos through tetraploid complementation can fix the non‐canonical imprinting defects in the TE of SCNT embryos.

### 2.4 Combined Overcoming SCNT Epigenetic Barriers for Pre‐ and Post‐Implantation Development Achieves a 30% Full‐Term Rate

**Figure 5:**
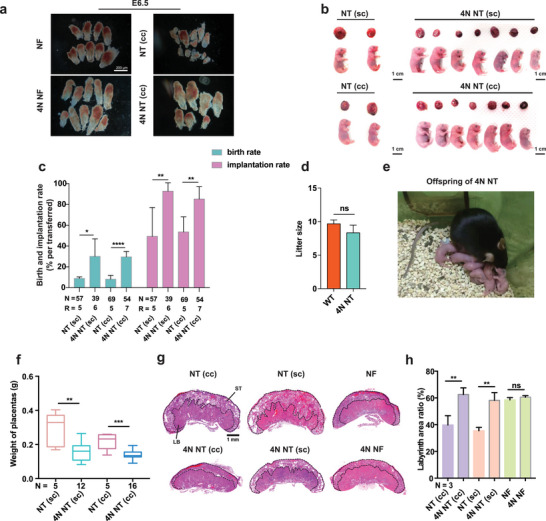
The combination of the cocktail method and TE replacement achieves efficient SCNT. a) Images showing E6.5 embryos of NF, 4N NF, NT (cc), and 4N NT (cc) embryos. “cc”: cumulus cell. Scale bars as presented. b) Newborn pups and corresponding placentas of NT (sc), 4N NT (sc), NT (cc), and 4N NT (cc) groups. “sc”: Sertoli cell. Scale bars, 1 cm. c) Bar graph showing the full‐term and implantation rates of NT (sc), 4N NT (sc), NT (cc), and 4N NT (cc) embryos. “N”, number of embryo transfers. “R”, number of recipients. Statistical analysis was done using Student's *t*‐test. ^****^ *p* < 0.0001, ^**^ *p* < 0.01, ^*^ *p* < 0.05, “ns”, not significant. d) Bar graph showing the litter size of WT and 4N NT parents. Statistical analysis was done using Student's *t*‐test. “ns”, not significant. e) The offspring of a 4N NT parent. f) Box plot showing the weight of E19.5 placentas from NT (sc), 4N NT (sc), NT (cc), and 4N NT (cc) embryos. Statistical analysis was done using Student's *t*‐test. ^***^ *p* < 0.001, ^**^ *p* < 0.01. The middle line in each box represents the median. Box edges and whiskers represent the 25th/75th and 2.5th/97.5th percentiles, respectively. g) Histological sections of E19.5 placentas stained with hematoxylin and eosin. The dashed lines outline the labyrinthine layer (LB) and the spongiotrophoblast layer (ST). Scale bar, 1 mm. h) Bar graph showing the area ratio of labyrinthine layer in histological sections. Statistical analysis was done using Student's *t*‐test. ^**^ *p* < 0.01, “ns”, not significant. Error bars represent SD.

We next examined the developmental efficiency of 4N and NT‐ICM reconstructed embryos. Sertoli cells (sc) and cumulus cells (cc) were used as male and female donor cells, respectively. SCNT was performed with the TSA, *Kdm4b*, and *Kdm5b* cocktail method treatment. Then, ICMs were isolated from these SCNT blastocysts and used for tetraploid complementation. The reconstructed embryos were cultured in vitro for 24 h to form blastocysts and then transferred to surrogate mothers. SCNT embryos (treated with the cocktail method) without TE replacement were used as controls. Embryos recovered at E6.5 showed better morphology for 4N NT embryos compared to NT embryos (**Figure** **5**a). The full‐term rate of NT (sc) with cocktail method treatment was 8.96% ± 1.39%, and the full‐term rate of NT (cc) with cocktail method treatment was 8.18% ± 3.58% (Figure 5b,c; Table S4, Supporting Information), which is similar to the rate reported in earlier studies to improve SCNT efficiency through overcoming epigenetic barriers for pre‐implantation development.^[^ ^16^, ^17^ ^]^ Importantly, the full‐term rate of 4N NT (sc) embryos was 30.17% ± 16.80%, and the full‐term rate of 4N NT (cc) embryos was 29.67% ± 5.07% (Figure 5b,c; Figure S9a, Table S4, Supporting Information). Compared to SCNT embryos, the implantation rate increased significantly to 92.75% ± 8.05% for 4N NT (sc) embryos and 85.2% ± 11.88% for 4N NT (cc) embryos (Figure 5c; Table S4, Supporting Information). Furthermore, 4N NT pups grew to adulthood normally and produced offspring by natural mating (Figure 5d,e; Figure S9b,c, Supporting Information).

Enlarged placentas with structural and functional defects are common in all cloned mammals and contribute to post‐implantation developmental defects in SCNT embryos.^[^ ^7^, ^47^, ^48^, ^49^, ^50^ ^]^ The placental weight of 4N NT embryos was significantly lower than that of NT embryos (Figure 5f; Table S4, Supporting Information). Nevertheless, there was no significant difference in body weight between NT and 4N NT pups (Figure S9d, Table S4, Supporting Information). Enlarged placentas in cloned animals are associated with an enlarged spongiotrophoblast (ST) layer and irregular spongiotrophoblast‐labyrinthine (LB) cell boundaries.^[^ ^34^, ^47^, ^48^, ^51^ ^]^ In line with this, the ST regions of NT (cc) and NT (sc) placentas were enlarged with more white vacuoles compared to NF and 4N NF placentas, as revealed by hematoxylin‐eosin staining (Figure 5g). In addition, the boundaries between the LB region and the ST region were irregular, and the labyrinth area ratio was significantly reduced in NT (cc) and NT (sc) placentas (Figure 5g,h). Interestingly, these defects in NT (cc) and NT (sc) placentas were largely absent in 4N NT (cc) and 4N NT (sc) placentas (Figure 5g,h).

Taken together, these results reveal that fixing the imprinting defects in the placenta by replacing the TE of SCNT embryos largely resolves the post‐implantation developmental defects of SCNT embryos. Overcoming both epigenetic barriers for pre‐implantation and post‐implantation development greatly improves the full‐term developmental rate of mouse cloning.

## 3 Discussion

In this study, we developed a strategy that involves *Kdm4d*, *Kdm5b*, and TSA treatment (“cocktail method”) of SCNT embryos, followed by TE replacement, to overcome key epigenetic barriers for SCNT embryos. These barriers include aberrant H3K9me3, H3K4me3, and histone acetylation, which impede pre‐implantation development, as well as loss of H3K27me3‐mediated non‐canonical imprinting that impedes post‐implantation development. Notably, our strategy achieves a cloning efficiency of ≈30% for both cumulus cells and Sertoli cells, a significant improvement over the previously reported best efficiency of 23.5%.^[^ ^25^ ^]^ The cocktail method significantly improved both the blastocyst rate and number of ICM cells in SCNT blastocysts, indicating that both lower blastocyst rate and lower number of ICM cells are the consequence of defective early embryonic development caused by epigenetic barriers, although the detailed mechanism awaits study in the future. Moreover, more than 300 DEGs remain in the ICMs of both cocktail‐treated SCNT embryos and reconstructed 4N NT embryos, suggesting that additional epigenetic barriers exist in the ICM lineage, which warrant further investigations in the future. Indeed, a recent report demonstrated aberrant H3K27me3 and H3K36me3 in SCNT pre‐implantation embryos,^[^ ^52^ ^]^ which may have contributed to the remaining DEGs observed here.

Replacing trophoblast cells in the cloned blastocysts by tetraploid complementation has been shown to be able to improve placental development of cloned embryos and increase cloning efficiency from around 2.7% to around 15.7%.^[^ ^39^ ^]^ However, the molecular mechanism underlying this approach has not been clear. Our study provides direct evidence that this approach fixes the loss of H3K27me3‐mediated non‐canonical imprinting in reconstructed SCNT embryos. In combination with previous findings that loss of H3K27me3‐mediated imprinting contributes to placental development defects and post‐implantation development failure in SCNT embryos,^[^ ^28^ ^]^ we conclude that H3K27me3‐mediated imprinting defects in SCNT embryos are fixed by TE replacement, leading to greatly improved post‐implantation development. One previous report showed that aggregating one *Kdm4b* and *Kdm5b* treated 4‐cell SCNT embryo with two 4‐cell 4N embryos increased the full‐term rate from 11.1% to 15.5%.^[^ ^17^ ^]^ This effect is relatively smaller compared to the result seen in our study, suggesting that the loss‐of‐imprinting TE cells from the SCNT embryo negatively affect post‐implantation development of reconstructed SCNT embryos. In contrast, this issue is absent in reconstructed embryos using SCNT ICMs in this study.

Most mechanistic studies on epigenetic barriers and strategies to improve cloning efficiency have focused on cumulus cells and Sertoli cells, including this study. However, cloning efficiency varies depending on the donor somatic cell type.^[^ ^4^, ^25^, ^53^, ^54^, ^55^, ^56^ ^]^ Cumulus cells and Sertoli cells generally exhibit relatively high cloning efficiency in mice. This discrepancy in cloning efficiency for different donor cell types suggests that additional cell‐type‐specific epigenetic barriers exist, warranting further investigation in the future. For example, fibroblasts, which are easy to obtain from large animals and humans, have been widely used as donor cells for cloning.^[^ ^12^, ^13^, ^57^, ^58^, ^59^ ^]^ However, they show relatively low cloning efficiency in mice for unknown reasons.^[^ ^25^, ^28^, ^60^, ^61^ ^]^ Understanding the epigenetic mechanisms underlying the low cloning efficiency of diverse types of cells could provide insights into how to improve cloning from these cell types. Our strategy here can serve as a reference for overcoming known epigenetic barriers.

Placental defects are common in cloned mammals^[^ ^7^, ^47^, ^49^, ^50^, ^51^, ^62^, ^63^, ^64^ ^]^ and are considered one of the major causes of low cloning efficiency. H3K27me3‐mediated imprinting for genes like *Sfmbt2*, *Jade1*, *Gab1*, *Smoc1*, *Slc38a4*, and *Gm32885* is critical for post‐implantation development, especially for placenta development and function.^[^ ^27^, ^28^, ^29^, ^32^, ^65^ ^]^ This study, along with previous studies, demonstrates that the loss of H3K27me3‐mediated non‐canonical imprinting is the main cause of placental defects in cloned mice. Mimicking H3K27me3‐mediated non‐canonical imprinting through heterozygous knockout in donor cells can fix these placental defects of SCNT embryos.^[^ ^27^, ^28^, ^29^ ^]^ However, whether H3K27me3‐mediated non‐canonical imprinting is a conserved mechanism across mammals is not fully understood.^[^ ^66^, ^67^ ^]^ Additionally, if this non‐canonical imprinting mechanism is conserved in other mammals, the genes imprinted by it are likely species‐specific.^[^ ^68^ ^]^ Therefore, the strategy of overcoming the loss of H3K27me3‐mediated non‐canonical imprinting barrier by mimicking imprinting through heterozygous knockout is practically impossible before fully understanding the molecular details of species‐specific H3K27me3‐mediated non‐canonical imprinting in these species. Moreover, mimicking imprinting through heterozygous knockout of non‐canonical imprinting genes introduces genetic changes into the donor cells, which are undesired, since cloning is intended to produce genetically identical organisms. However, these challenges can be bypassed using the strategy presented in this study, which involves TE replacement through embryo micromanipulation, without requiring detailed knowledge of species‐specific imprinting in the placenta. Therefore, we anticipate that this strategy will be easily adaptable and widely applicable for improving cloning efficiency in other mammals.

## 4 Experimental Section

### Mice

B6D2F1 (C57BL/6 female × DBA2 male) female mice at 8–12 weeks were used as oocyte recipients for SCNT. Cumulus cells (female) and Sertoli cells (male) were collected from B6D2F1 background mice. For testing the full‐term rate of reconstructed embryos, B6D2F1 females were superovulated and mated with B6D2F1 males to generate 2‐cell embryos for the preparation of tetraploid embryos. For the preparation of TE samples for RNA‐seq, C57BL/6 females (Zhejiang Vital River, No. 213) were superovulated and mated with DBA2 males (Zhejiang Vital River, No. 214) to generate fertilization‐derived embryos and 2‐cell embryos for preparation of tetraploid embryos, and the SCNT embryos were prepared from cumulus cells in the B6D2F1 background. Adult pseudopregnant ICR females (Zhejiang Vital River, No. 201) were used as recipients for embryo transplantation. In the 4N cell tracing experiment, C57BL/6^GFP^ mice (GemPharmatech, T006163) were used to carry the fluorescent reporter. C57BL/6^GFP^ females were superovulated and mated with DBA2 males to generate fertilized embryos and 2‐cell embryos for the preparation of tetraploid embryos with GFP reporter. Cumulus cells were collected from B6D2F1^GFP^ (C57BL/6^GFP^ female × DBA2 male) female mice at 8–12 weeks and used as donor cells for fluorescent SCNT embryo preparation. All mice were housed under constant temperature, humidity, ventilation, and automatic light cycles in the specific pathogen‐free animal facility of the Center for Excellence in Brain Science and Intelligence Technology (Institute of Neuroscience), Chinese Academy of Sciences.

### Ethics

All the animal experimental procedures were approved (NA‐041‐2019) by the Institutional Animal Care and Use Committee (IACUC) of the Center for Excellence in Brain Science and Intelligence Technology (Institute of Neuroscience), Chinese Academy of Sciences.

### Donor Cell Preparation

Cumulus cells were collected from 8 to 12‐week‐old B6D2F1 female mice. The mice were superovulated by injecting 5 IU of pregnant mare serum gonadotropin (PMSG) and 5 IU of human chorionic gonadotropin (hCG) at intervals of 48 h. 14 h after hCG injection, cumulus‐oocyte complexes (COCs) were collected from the oviducts and then treated with bovine testicular hyaluronidase (Sigma, H3884) to obtain dissociated cumulus cells and oocytes.

Sertoli cells were collected from the testes of 3–5‐day‐old B6D2F1 male mice as previously described.^[^ ^56^ ^]^ Testicular masses were incubated in PBS containing 0.1 mg mL^−1^ collagenase for 30 min at 37 °C followed by a 5‐min treatment in 0.25% Trypsin with 1mM EDTA at room temperature. The cells were further washed three times with HCZB to obtain dissociated Sertoli cells.

### Somatic Cell Nuclear Transfer and RNA Injection

All the oocytes and embryos were cultured in an incubator at 37 °C with 5% CO_2_. For mouse SCNT, mouse MII oocytes from B6D2F1 mice were collected 14 h after hCG injection and transferred to HCZB manipulation drops with 5 µg mL^−1^ cytochalasin B (Sigma, C6762) in a glass‐bottom dish. The spindle‐chromosome complex was removed rapidly by a piezo‐driven pipette. The enucleated oocytes were transferred to pre‐equilibrated KSOM medium (Sigma, MR‐106‐D) at 37 °C under 5% CO_2_. After all oocytes were enucleated, cumulus cells or Sertoli cells were transferred into enucleated oocytes by direct injection. Reconstructed embryos were activated in Ca^2+^ free CZB medium with 10 mm SrCl_2_, 5 µg mL^−1^ cytochalasin B, and 10 nm TSA (Sigma, T1952). For *Kdm4d* and *Kdm5b* mRNA injection, about 10 pl of *Kdm4d* and *Kdm5b* mRNA at 1000 ng µL^−1^ (500 ng µL^−1^ each) were injected 5 h after activation with a piezo‐driven micromanipulator. Reconstructed embryos were cultured in KSOM medium with 10 nm TSA for another 5 h. The embryos were then transferred to KSOM medium for further development.

### In Vitro Transcription of mRNA

Mouse cDNAs for *Kdm4d* (Forward: TAATACGACTCACTATAGGGAGAcagtgaattcgagctcggtacct, Reverse: GCCCTCTAGACTCGAGGTACGC) and *Kdm5b* (Forward: TAATACGACTCACTATAGGGAGAatggagccggccaccg, Reverse: TTACTTTCGGCTTGGTGCGTCC) were cloned into vectors with a T7 promoter. The PCR products were purified by the QIAquick PCR Purification Kit (QIAGEN, 28104). In vitro transcription was performed with the mMESSAGE mMACHINE T7 Ultra Kit (Life Technologies, AM1345) according to the manufacturer's instructions. In vitro transcribed mRNA was purified by the MEGAclear Kit (Life Technologies, AM1908). The concentration of each mRNA was adjusted to 1000 ng µL^−1^ before storing at −80 °C. The *Kdm4d* and *Kdm5b* mRNA were mixed 1:1 before injection.

### Immunofluorescence Staining

Blastocysts were collected and fixed in 4% paraformaldehyde in PBS for 30 min at room temperature. The embryos were then thoroughly rinsed with PBS to remove any excess fixative. Next, they were permeabilized using 0.1% Triton X‐100 in PBS for 10–15 min at room temperature, followed by additional PBS washes. To block non‐specific binding, the embryos were incubated in 5% BSA (Sigma, A1933) for 1 h at room temperature. Subsequently, the embryos were incubated overnight at 4 °C with the primary antibody diluted in the blocking solution. After this incubation, the embryos were washed several times in PBS to remove unbound antibody. The embryos were then incubated with a fluorescently conjugated secondary antibody for 1 h at room temperature in the dark, followed by thorough PBS rinsing. DAPI was used to visualize cell nuclei. The stained embryos were imaged using an Olympus FV10i microscope.

### Preparation of Tetraploid Embryos

2‐cell stage embryos were collected 44 h after hCG injection from the oviducts. The 2‐cell embryos were electrofused to produce tetraploid 1‐cell embryos. In detail, the 2‐cell embryos were aligned using an alternating current in 0.3 m mannitol solution (Sigma, M4125) with 0.3% BSA, and a single direct current pulse of 30 V was applied for 40 µs. After electrofusion, the embryos were returned to KSOM medium, and the fused tetraploid embryos were cultured for one day to reach the 4‐cell stage.

### ICM Isolation from Blastocysts

ICM was isolated by immunosurgery as described previously.^[^ ^69^ ^]^ The zona pellucida of cloned or fertilization blastocysts was removed by acid Tyrode's solution treatment (Sigma, C6762). Then the blastocysts were incubated with rabbit anti‐mouse serum (Sigma, M5774, diluted 1:1 with KSOM) for 30 min. After thorough washes with M2 (Sigma, M7176) over 20 times, blastocysts were exposed to guinea pig serum (Sigma, 234395, diluted 1:1 with KSOM) for another 30 min. The outer TE cells were lysed within a few minutes. The dead TE cells were removed by gently pipetting the ICMs in a glass pipette with a diameter of 40–60 µm after an additional 1–3 h culturing in KSOM.

### TE Isolation from Blastocysts

After the removal of zona pellucida from blastocysts, TE was obtained by mechanical micromanipulation with laser assistance. The blastocyst was held by a glass pipette on the polar trophoblast near the ICM and cut with the laser. Another glass pipette was used to suck on the mural trophoblast away from ICM and pull outward to get isolated TE without ICM contamination.

### Aggregation of Isolated ICM and Tetraploid 4‐Cell Embryos

The aggregation plate was prepared as described^[^ ^70^ ^]^ using an aggregation needle. The zona pellucida of the tetraploid embryos was removed by acid Tyrode's solution treatment. Two 4‐cell stage tetraploid embryos were placed into each of the depressions in the aggregation plate, and one ICM from SCNT or a fertilized embryo was placed into one depression. Embryos in each depression should be physically contacted. The aggregates were cultured in a humidified incubator with 5% CO_2_ at 37 °C for 24–36 h to reach the blastocyst stage.

### Embryo Transfer and Full‐Term Rate Analysis

Successfully aggregated embryos, fertilization‐derived embryos, and SCNT embryos were transferred into the uterine horns of 2.5‐day‐postcoitum (dpc) pseudopregnant ICR females mated with vasectomized males. On day 19.5 of gestation, the recipient females were subjected to cesarean section, and live pups were nursed by lactating ICR females. Live pups and placentas were photographed, and the weight of pups and placentas was recorded. For full‐term rate analysis, each group had at least two biological replicates. The full‐term rate was calculated by dividing the number of newborn pups by the number of embryos transferred.

### H&E Staining

Term placentas (E19.5) were fixed with Bouin's solution and processed for paraffin sectioning. Sections with a thickness of 4 µm were stained with hematoxylin and eosin as previously described.^[^ ^29^ ^]^

### FACS Sorting

The E9.5 fetuses and placentas were collected and then cut into ≈0.5 mm pieces, which were transferred into 15 mL tubes. The tissue was then treated with 0.25% Trypsin‐EDTA at 37 °C for 10 min on a shaker set to 200 rpm. After incubation, 2 mL of DMEM medium containing 10% FBS was added to halt the dissociation, and the mixture was pipetted several times to ensure complete disaggregation. Following filtration, GFP‐positive cells were analyzed using a BECKMAN MoFlo Astrios Cell Sorter.

### RNA‐seq Library Preparation

For each replicate of RNA‐seq experiments, the sample was lysed directly in 1× lysis buffer containing RNase inhibitor. cDNA synthesis and PCR amplification were performed using SMART‐Seq v4 Ultra Low Input RNA Kit for Sequencing (Clontech, 634888) according to the manufacturer's instructions. After PCR amplification, the sequencing libraries were made from the amplified cDNA using TruePrep DNA Library Prep Kit V2 for Illumina according to the manufacturer's instructions (Vazyme, TD503). Libraries were quantified using a Qubit dsDNA HS Assay Kit (Thermo Scientific, Q32854), quality‐controlled using a Fragment Analyzer instrument (Agilent). Libraries were subjected to pair‐end 150 bp sequencing on a NovaSeq 6000 sequencer (Illumina).

### RNA‐seq Data Analysis

The raw sequencing reads were trimmed using fastp with default parameters to remove low‐quality bases and adapters in paired‐end reads.^[^ ^71^ ^]^ The remaining reads were mapped to the mouse genome (mm10) with parameters “‐no‐mixed, ‐no‐discordant”. Raw read counts for each gene were calculated by FeatureCounts^[^ ^72^ ^]^ in the Subread package. Expression levels of each gene were quantified as normalized TPM (transcripts per million mapped fragments). Differential gene expression analysis was performed using the DESeq2 package in the Bioconductor R program.^[^ ^73^ ^]^ The cutoffs used for the analyses were specified in the figure legends. The Pearson correlation coefficient of gene expression level was calculated to quantify the correlation between replicates. For SNP tracking, parental SNP information was used to determine the parental origin assignment of RNA‐seq data from the hybrid embryos. The SNP information between C57BL/6 and DBA2 was downloaded from the Mouse Genome Project (ftp://ftp‐mouse.sanger.ac.uk/REL‐1211‐SNPs_Indels/), and only high‐quality homozygous SNPs were included in the analysis. The SNPsplit software was used to split mapped reads to their paternal origin.^[^ ^74^ ^]^

### Biological Replicates

The number of biological replicates for each analysis was indicated in the relevant Figure legends and Results. Briefly, three blastocysts were used for immunostaining for each of the sample, 39–69 blastocysts were transferred to pseudopregnant female mice to examine postimplantation development (the number of pseudopregnant females is shown in Table S4, Supporting Information), 5–16 placentae were examined for their weight and histology, 2 pooled samples were used for RNA‐seq.

### Statistics and Reproducibility

No statistical methods were used to pre‐determine sample sizes, but the sample sizes were similar to those used in previous publications.^[^ ^16^, ^25^, ^26^, ^28^ ^]^ The oocytes and embryos were randomly assigned to each experimental group. Data collection and analysis were not performed blindly to the conditions of the experiments. No data were excluded from the analysis. The data analysis and graph were generated by GraphPad Prism. All mean values are the mean ± SD. The accurate values of N and R are shown in each figure legend. The *p*‐values are also shown in each figure legend.

## Conflict of Interest

Y.L., Zhen.L., Q.S., and F.L. are named inventors on a patent filed by the Center for Excellence in Brain Science and Intelligence Technology and the Institute of Genetics and Developmental Biology covering the SCNT strategy developed in this study. The other authors declare no competing interests.

## Author Contributions

Y.L., S.S., Yuting.X., and J.Z. contributed equally. F.L., Q.S., and Zhen.L. conceived and supervised the project. F.L., Q.S., Zhen.L., and Y.L. designed the experiments. S.S. and Yuting.X. performed SCNT with the help of Zhaodi.L., J.Z., Y.D., Y.C., Yali.X., and M.W. performed RNA‐seq and bioinformatic analysis. X.B. performed H&E staining. Y.L. performed most of the other experiments with help from Y.D. and J.H. Y.L. and F.L. wrote the manuscript with the input of the other authors.

## Supporting information



Supporting Information

## Data Availability

RNA‐seq data generated in this study have been deposited in the Genome Sequence Archive (GSA: https://ngdc.cncb.ac.cn/gsa/) hosted by the National Genomics Data Center under the accession number CRA023383.
