# Supplementary material for: Efficient Somatic Cell Nuclear Transfer by Overcoming Both Pre‐ and Post‐Implantation Epigenetic Barriers
Source: Adv Sci (Weinh). 2025 Jul 8;12(37):e04669. doi: 10.1002/advs.202504669 (PMC12499444; doi:10.1002/advs.202504669)
Supplement: Supplementary file 1 — Supporting Information [file ADVS-12-e04669-s001.pdf]

ADVANCED SCIENCE
Open Access

Supporting Information

for *Adv. Sci.*, DOI 10.1002/advs.202504669

Efficient Somatic Cell Nuclear Transfer by Overcoming Both Pre- and Post-Implantation Epigenetic Barriers

Yamei Li, Shiyu Sun, Yuting Xu, Jixiang Zhang, Yi Du, Yaxin Cao, Zhaodi Liao, Yali Xie, Xinyan Bian, Jiantao Huang, Meijiao Wang, Zhen Liu, Qiang Sun* and Falong Lu**

Supporting Information

Efficient somatic cell nuclear transfer by overcoming both pre- and post-implantation epigenetic barriers

Yamei Li[1,2,3,5], Shiyu Sun[2,3,5], Yuting Xu[3,5], Jixiang Zhang[1,2,5], Yi Du[1,2], Yaxin Cao[1], Zhaodi Liao[2,3], Yali Xie[1,2], Xinyan Bian[3], Jiantao Huang[3], Meijiao Wang[1], Zhen Liu[2,3,4,#], Qiang Sun[2,3,4,#], Falong Lu[1,2,#]

[1]State Key Laboratory of Molecular Developmental Biology, Institute of Genetics and Developmental Biology, Chinese Academy of Sciences, Beijing 100101, China.

[2]University of Chinese Academy of Sciences, Beijing 100049, China.

[3]Institute of Neuroscience, CAS Key Laboratory of Primate Neurobiology, State Key Laboratory of Neuroscience, Center for Excellence in Brain Science and Intelligence Technology, Chinese Academy of Sciences, Shanghai, 200031, China.

[4]Shanghai Center for Brain Science and Brain-Inspired Intelligence Technology, Shanghai 201210, China.

[5]These authors contributed equally.

[#] Correspondence:

zliu2010@ion.ac.cn (Zhen.L.), qsun@ion.ac.cn (Q.S.), fllu@genetics.ac.cn (F.L.)

Figure S1

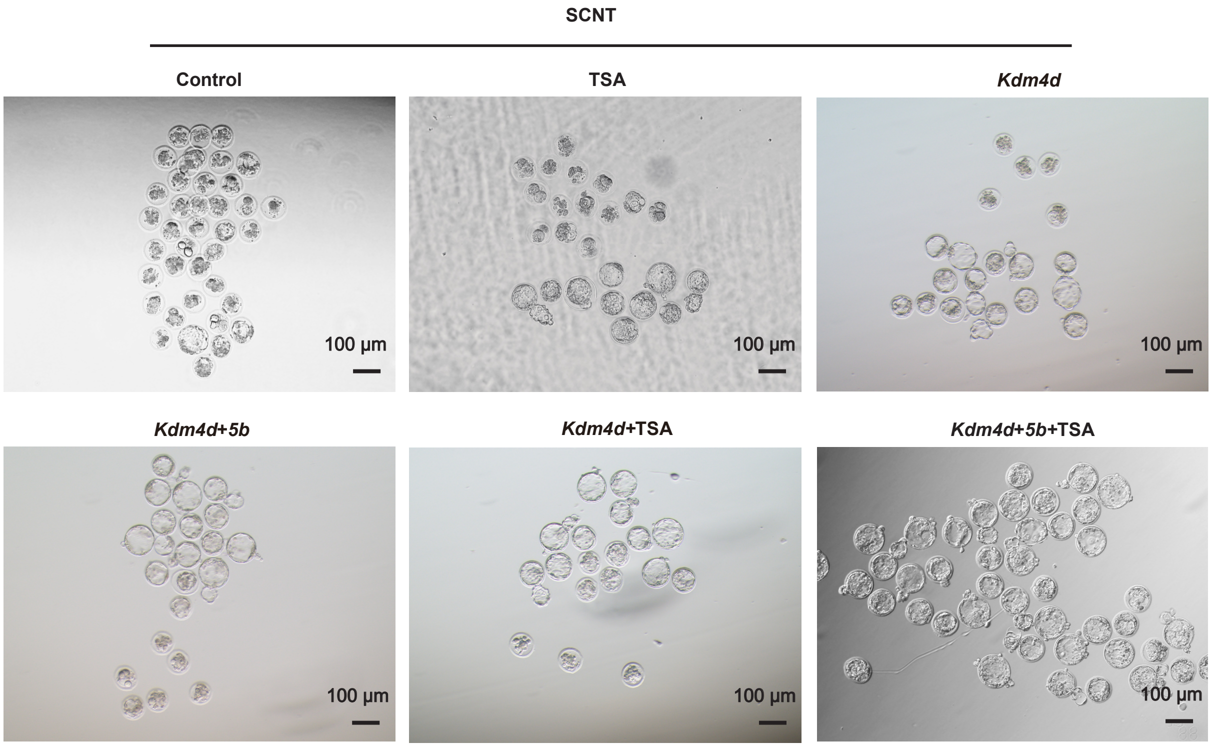
SCNT
Control
TSA
Kdm4d
Kdm4d+5b
Kdm4d+TSA
Kdm4d+5b+TSA
100 µm
100 µm
100 µm
100 µm
100 µm
100 µm

Figure S1. TSA treatment combined with *Kdm4d* and *Kdm5b* mRNA injection increases blastocyst rate of SCNT embryos.

Images of SCNT blastocysts and arrested SCNT embryos in Control, TSA treated, *Kdm4d* treated, *Kdm4d+5b* treated, *Kdm4d*+TSA treated, and *Kdm4d+5b*+TSA treated groups. Scale bars, 100 μm.

Figure S2

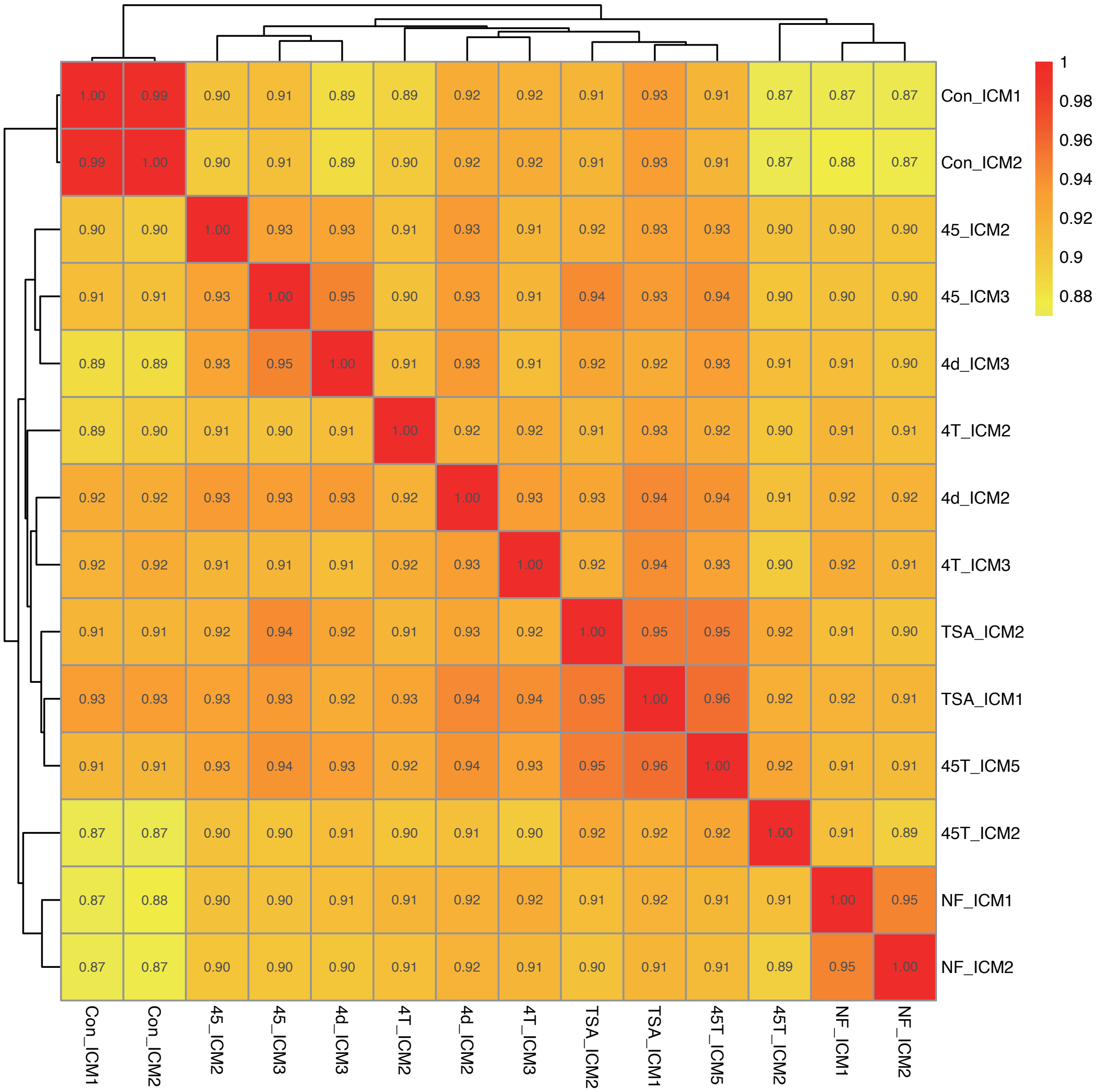
Con_ICM1
Con_ICM2
45_ICM2
45_ICM3
4d_ICM3
4T_ICM2
4d_ICM2
4T_ICM3
TSA_ICM2
TSA_ICM1
45T_ICM5
45T_ICM2
NF_ICM1
NF_ICM2
1
0.98
0.96
0.94
0.92
0.9
0.88

Figure S2. Transcriptomes of ICM samples.

Correlation heatmap showing the correlation between NF ICM and SCNT ICMs in Control, TSA treated, *Kdm4d* treated, *Kdm4d+5b* treated, *Kdm4d*+TSA treated, and *Kdm4d+5b*+TSA treated groups.

Figure S3

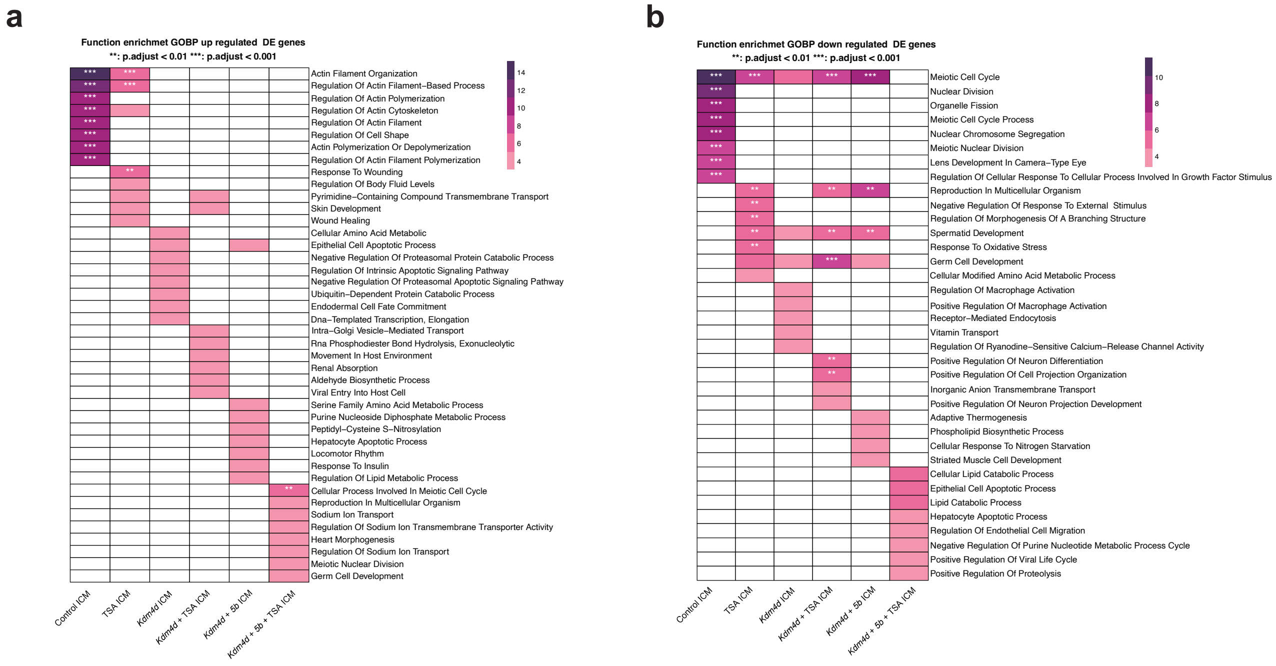
a
Function enrichmet GOBP up regulated DE genes
**: p.adjust < 0.01 ***: p.adjust < 0.001
Actin Filament Organization
Regulation Of Actin Filament–Based Process
Regulation Of Actin Polymerization
Regulation Of Actin Cytoskeleton
Regulation Of Actin Filament
Regulation Of Cell Shape
Actin Polymerization Or Depolymerization
Regulation Of Actin Filament Polymerization
Response To Wounding
Regulation Of Body Fluid Levels
Pyrimidine–Containing Compound Transmembrane Transport
Skin Development
Wound Healing
Cellular Amino Acid Metabolic
Epithelial Cell Apoptotic Process
Negative Regulation Of Proteasomal Protein Catabolic Process
Regulation Of Intrinsic Apoptotic Signaling Pathway
Negative Regulation Of Proteasomal Apoptotic Signaling Pathway
Ubiquitin–Dependent Protein Catabolic Process
Endodermal Cell Fate Commitment
Dna–Templated Transcription, Elongation
Intra–Golgi Vesicle–Mediated Transport
Rna Phosphodiester Bond Hydrolysis, Exonucleolytic
Movement In Host Environment
Renal Absorption
Aldehyde Biosynthetic Process
Viral Entry Into Host Cell
Serine Family Amino Acid Metabolic Process
Purine Nucleoside Diphosphate Metabolic Process
Peptidyl–Cysteine S–Nitrosylation
Hepatocyte Apoptotic Process
Locomotor Rhythm
Response To Insulin
Regulation Of Lipid Metabolic Process
Cellular Process Involved In Meiotic Cell Cycle
Reproduction In Multicellular Organism
Sodium Ion Transport
Regulation Of Sodium Ion Transmembrane Transporter Activity
Heart Morphogenesis
Regulation Of Sodium Ion Transport
Meiotic Nuclear Division
Germ Cell Development
14
12
10
8
6
4
Control ICM
TSA ICM
Kdm4d ICM
Kdm4d + TSA ICM
Kdm4d + 5b ICM
Kdm4d + 5b + TSA ICM
b
Function enrichmet GOBP down regulated DE genes
**: p.adjust < 0.01 ***: p.adjust < 0.001
Meiotic Cell Cycle
Nuclear Division
Organelle Fission
Meiotic Cell Cycle Process
Nuclear Chromosome Segregation
Meiotic Nuclear Division
Lens Development In Camera–Type Eye
Regulation Of Cellular Response To Cellular Process Involved In Growth Factor Stimulus
Reproduction In Multicellular Organism
Negative Regulation Of Response To External Stimulus
Regulation Of Morphogenesis Of A Branching Structure
Spermatid Development
Response To Oxidative Stress
Germ Cell Development
Cellular Modified Amino Acid Metabolic Process
Regulation Of Macrophage Activation
Positive Regulation Of Macrophage Activation
Receptor–Mediated Endocytosis
Vitamin Transport
Regulation Of Ryanodine–Sensitive Calcium–Release Channel Activity
Positive Regulation Of Neuron Differentiation
Positive Regulation Of Cell Projection Organization
Inorganic Anion Transmembrane Transport
Positive Regulation Of Neuron Projection Development
Adaptive Thermogenesis
Phospholipid Biosynthetic Process
Cellular Response To Nitrogen Starvation
Striated Muscle Cell Development
Cellular Lipid Catabolic Process
Epithelial Cell Apoptotic Process
Lipid Catabolic Process
Hepatocyte Apoptotic Process
Regulation Of Endothelial Cell Migration
Negative Regulation Of Purine Nucleotide Metabolic Process Cycle
Positive Regulation Of Viral Life Cycle
Positive Regulation Of Proteolysis
10
8
6
4
Control ICM
TSA ICM
Kdm4d ICM
Kdm4d + TSA ICM
Kdm4d + 5b ICM
Kdm4d + 5b + TSA ICM

Figure S3. Gene Ontology (GO) analysis of differentially expressed genes (DEGs).

(a) Gene Ontology of biological processes (GOBP) analysis of upregulated DEGs in SCNT embryos (Control, TSA treated, *Kdm4d* treated, *Kdm4d+5b* treated, *Kdm4d*+TSA treated, and *Kdm4d+5b*+TSA treated). **: p.adjust < 0.01, ***: p.adjust < 0.001.

(b) GOBP analysis of downregulated DEGs in SCNT embryos (Control, TSA treated, *Kdm4d* treated, *Kdm4d+5b* treated, *Kdm4d*+TSA treated, and *Kdm4d+5b*+TSA treated). **: p.adjust < 0.01, ***: p.adjust < 0.001.

Figure S4

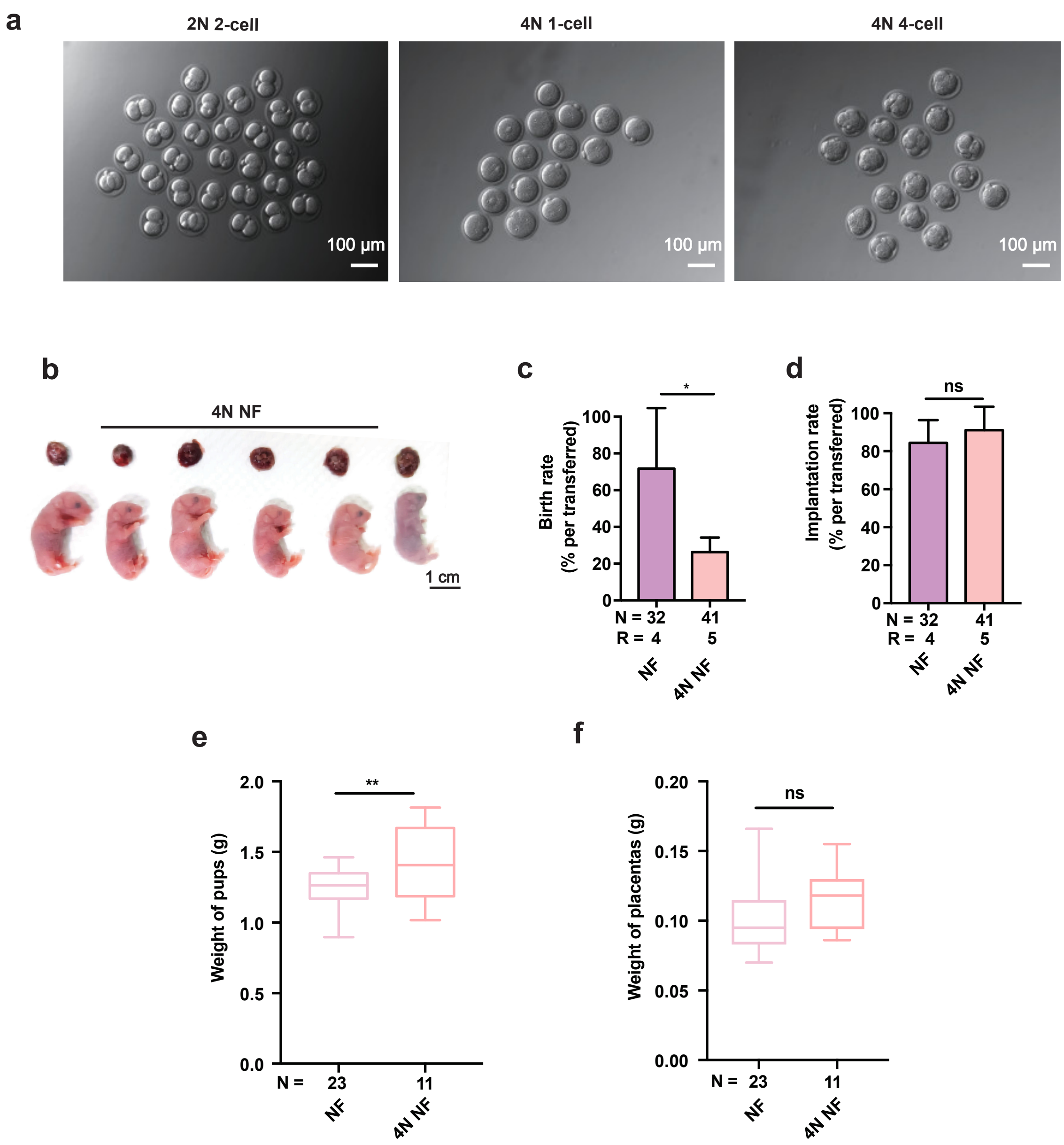

a
2N 2-cell
4N 1-cell
4N 4-cell
100 μm
100 μm
100 μm
b
4N NF
1 cm
c
*
Birth rate (% per transferred)
100
80
60
40
20
0
N = 32
41
R = 4
5
NF
4N NF
d
ns
Implantation rate (% per transferred)
100
80
60
40
20
0
N = 32
41
R = 4
5
NF
4N NF
e
**
Weight of pups (g)
2.0
1.5
1.0
0.5
0.0
N = 23
11
NF
4N NF
f
ns
Weight of placentas (g)
0.20
0.15
0.10
0.05
0.00
N = 23
11
NF
4N NF

Figure S4. Generation of 4N NF mouse.

(a) Images showing the production of tetraploid embryos. Scale bars, 100 μm.

(b) Images showing newborn pups and corresponding placentas from the 4N NF group. Scale bar, 1 cm.

(c-f) Bar graphs showing the birth rate **(c)** and implantation rate **(d)**, and box plots showing the weight of pups **(e)** and placentas **(f)** in NF and 4N NF groups. Statistical analysis was done using Student's *t*-test. ** $p < 0.01$, * $p < 0.05$, "ns", not significant. Error bars represent SD. In box plots, the middle line in each box represents the median. Box edges and whiskers represent the 25th/75th and 2.5th/97.5th percentiles, respectively.

Figure S5

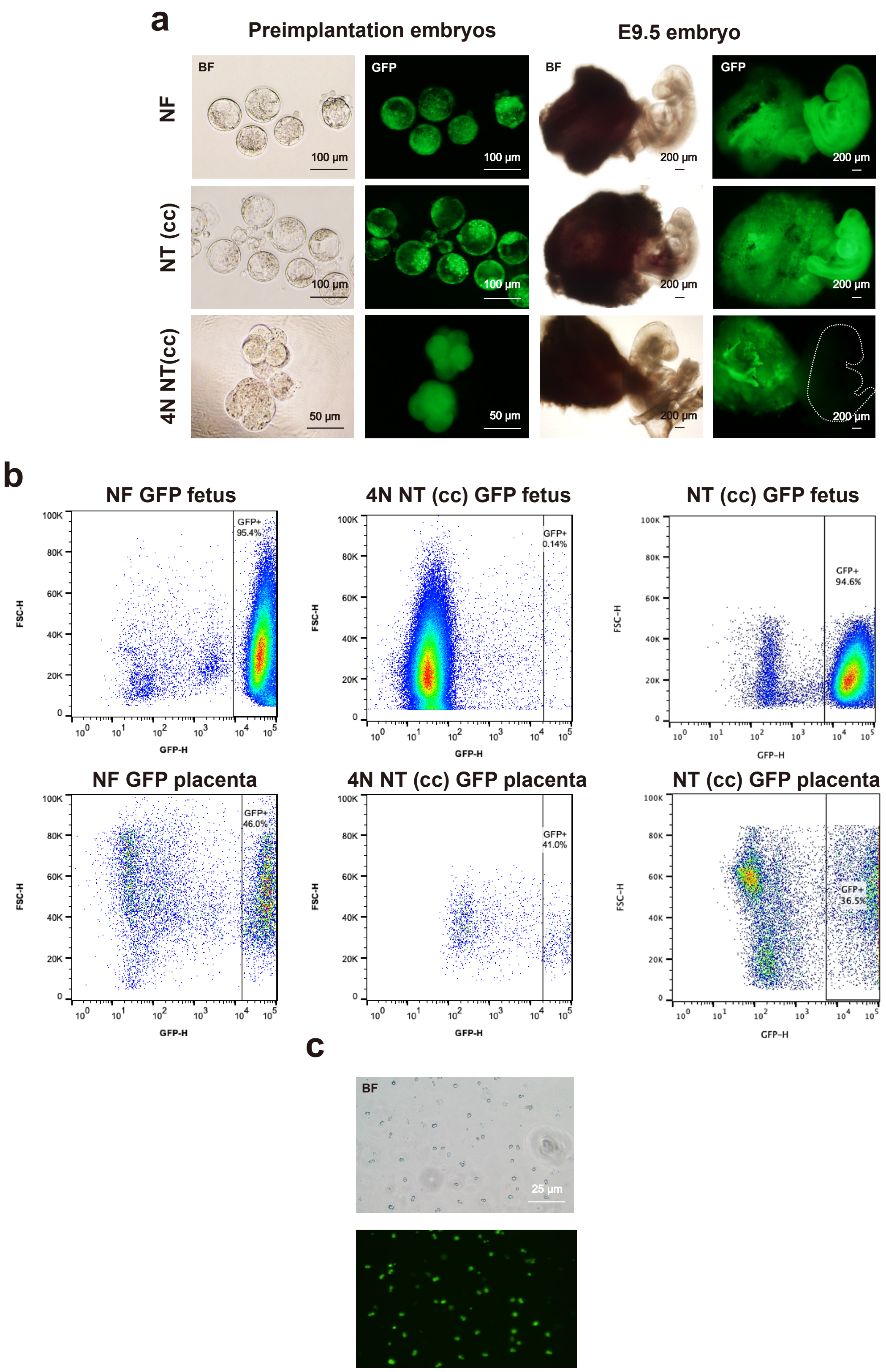
a
Preimplantation embryos
E9.5 embryo
BF
GFP
BF
GFP
NF
NT (cc)
4N NT(cc)
100 µm
50 µm
200 µm
b
NF GFP fetus
4N NT (cc) GFP fetus
NT (cc) GFP fetus
GFP+ 95.4%
GFP+ 0.14%
GFP+ 94.6%
NF GFP placenta
4N NT (cc) GFP placenta
NT (cc) GFP placenta
GFP+ 46.0%
GFP+ 41.0%
GFP+ 36.5%
FSC-H
GFP-H
c
BF
25 µm

Figure S5. Tetraploid cells contribute minimally to the fetus.

(a) Preimplantation and E9.5 NF, NT (cc), and 4N NT (cc) embryos. GFP reporter is from $B6^{GFP}$ female mouse. For NF and NT (cc) groups, the GFP reporter is present in the nuclear genome of the embryos. For the 4N NT group, the GFP reporter is present in the nuclear genome of the tetraploid embryos but not in the ICM of SCNT embryos. Scale bars as indicated in each image.

(b) FACS analysis of GFP-positive cells in NF, 4N NT (cc) and NT (cc) E9.5 fetuses and placentas.

(c) Visulization of GFP-positive cells after FACS.

Figure S6

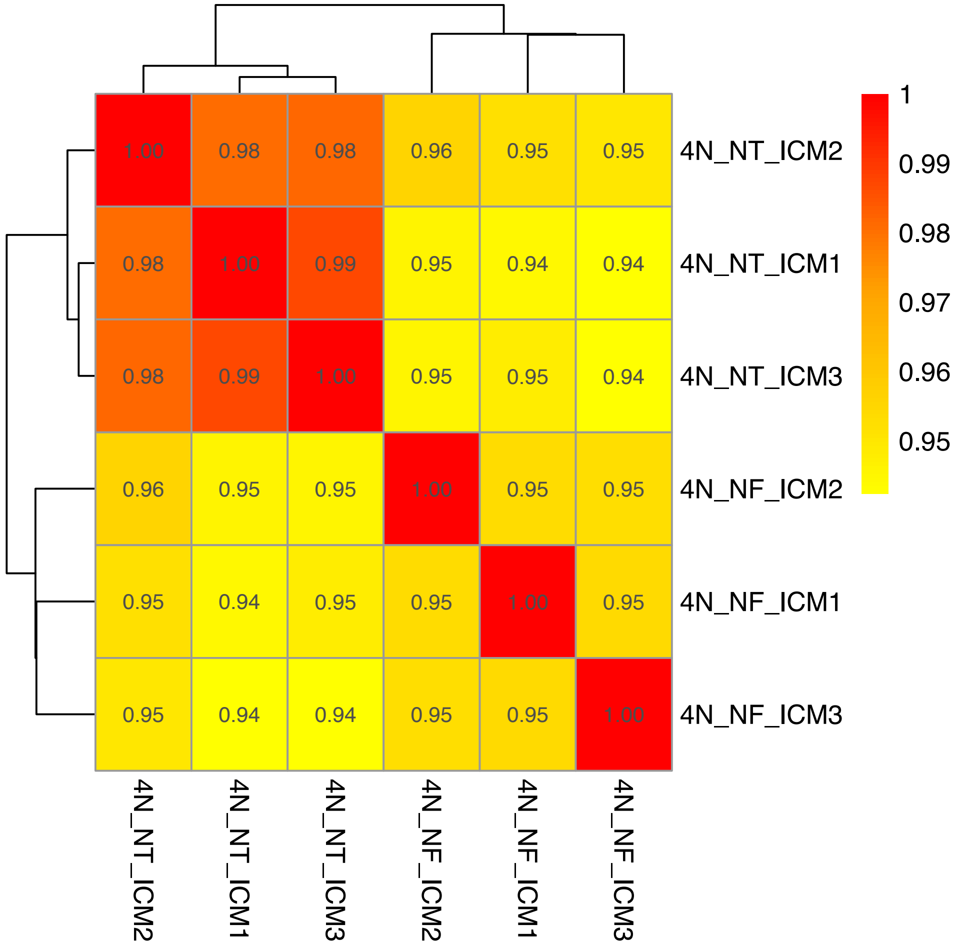

1.00
0.98
0.98
0.96
0.95
0.95
0.98
1.00
0.99
0.95
0.94
0.94
0.98
0.99
1.00
0.95
0.95
0.94
0.96
0.95
0.95
1.00
0.95
0.95
0.95
0.94
0.95
0.95
1.00
0.95
0.95
0.94
0.94
0.95
0.95
1.00
4N_NT_ICM2
4N_NT_ICM1
4N_NT_ICM3
4N_NF_ICM2
4N_NF_ICM1
4N_NF_ICM3
1
0.99
0.98
0.97
0.96
0.95

Figure S6. Transcriptomes of ICMs from 4N NT and 4N NF embryos.

Correlation heatmap showing the correlation between 4N NT ICM and 4N NF ICM groups.

Figure S7

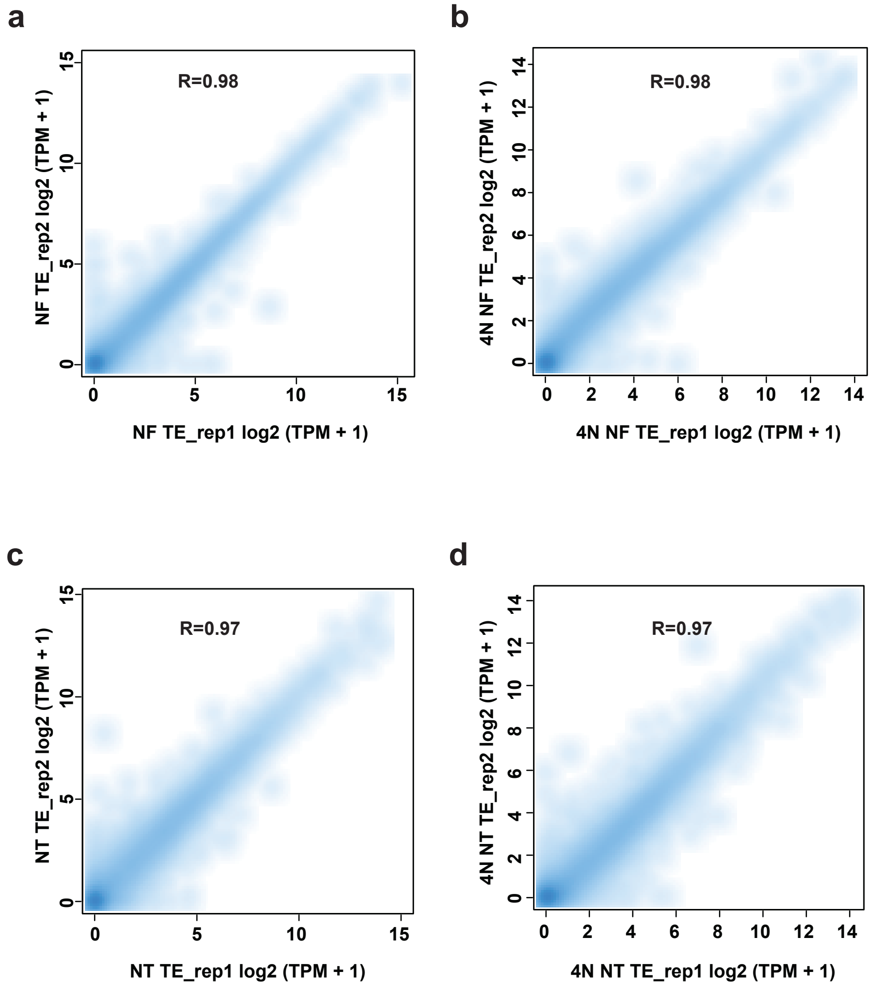

a
R=0.98
NF TE_rep2 log2 (TPM + 1)
NF TE_rep1 log2 (TPM + 1)
0
5
10
15
b
R=0.98
4N NF TE_rep2 log2 (TPM + 1)
4N NF TE_rep1 log2 (TPM + 1)
0
2
4
6
8
10
12
14
c
R=0.97
NT TE_rep2 log2 (TPM + 1)
NT TE_rep1 log2 (TPM + 1)
d
R=0.97
4N NT TE_rep2 log2 (TPM + 1)
4N NT TE_rep1 log2 (TPM + 1)

Figure S7. Transcriptomes of TE samples.

(a-d) Correlation analysis of RNA-seq biological replicates of TE samples from NF (**a**), 4N NF (**b**), NT (**c**), and 4N NT (**d**) groups. “R”, Pearson correlation coefficient.

Figure S8

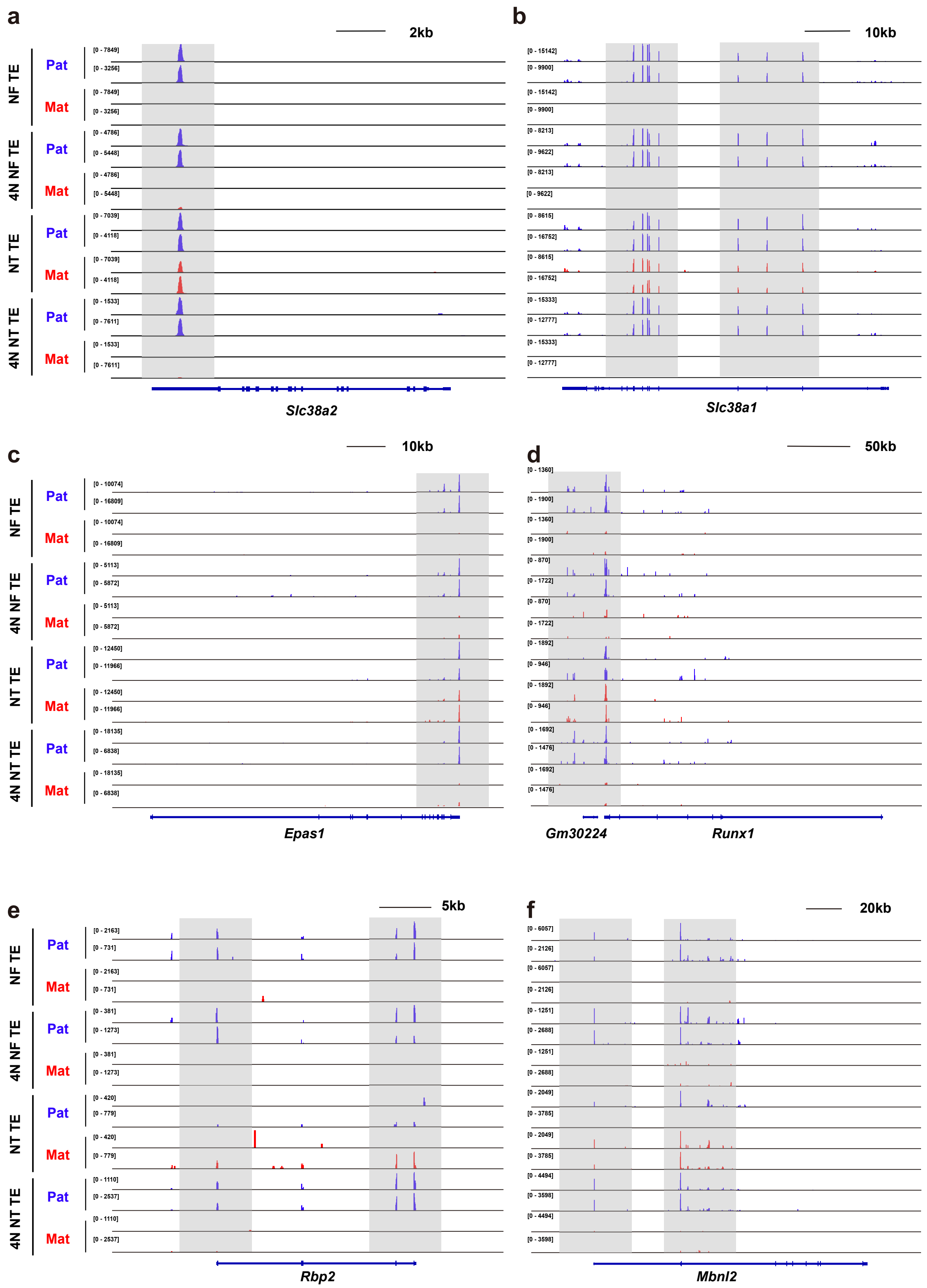
a
2kb
NF TE
4N NF TE
NT TE
4N NT TE
Pat
Mat
Slc38a2
b
10kb
Slc38a1
c
10kb
Epas1
d
50kb
Gm30224
Runx1
e
5kb
Rbp2
f
20kb
Mbnl2

Figure S8. Replacement of SCNT trophoblast by tetraploid complementation corrects defects in H3K27me3-mediated non-canonical imprinting in TEs.

(a-f) Genome browser views of RNA-seq data for H3K27me3-controlled non-canonical imprinting genes, including *Slc38a2*, *Slc38a1, Epas1, Runx1, Rbp2*, and *Mbnl2*. H3K27me3-controlled non-canonical imprinting genes are paternally expressed in NF, 4N NF, and 4N NT embryos, but biallelically expressed in NT embryos. Paternal expression is shown in blue, and maternal expression is shown in red. Scale bars are shown at the top of each view.

Figure S9

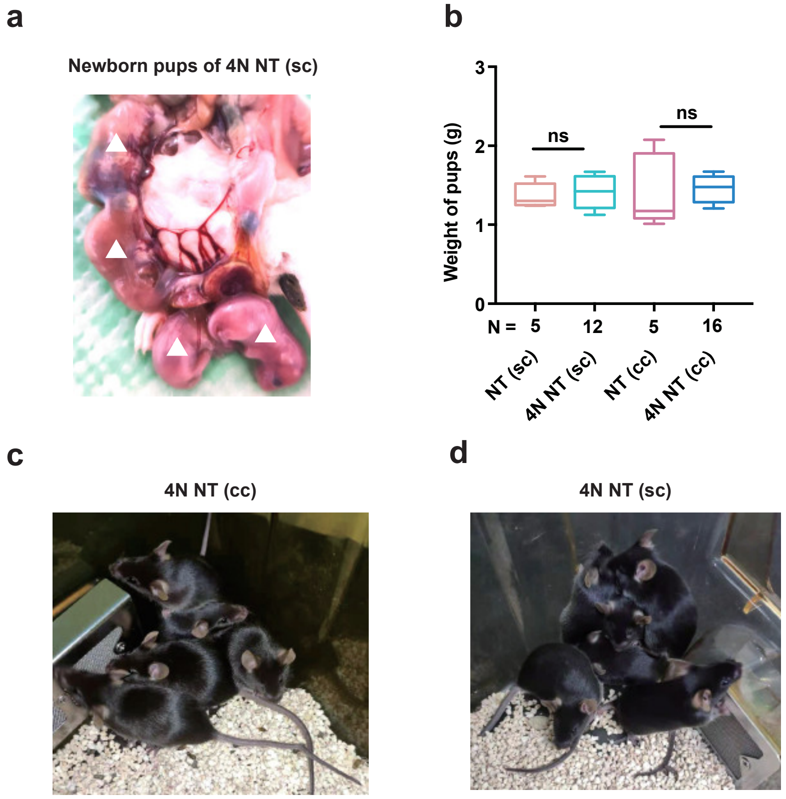
a
Newborn pups of 4N NT (sc)
b
Weight of pups (g)
0
1
2
3
ns
ns
N = 5
12
5
16
NT (sc)
4N NT (sc)
NT (cc)
4N NT (cc)
c
4N NT (cc)
d
4N NT (sc)

Figure S9. Cloned mice using the combination of the cocktail method and TE replacement strategy.

(a) Newborn pups of 4N NT (sc) mice from a surrogate mother. The white arrow indicated newborn pups.

(**b-c**) Images showing adult 4N NT (cc) and 4N NT (sc) mice.

(d) Box plot showing the weight of newborn pups from NT (sc), 4N NT (sc), NT (cc), and 4N NT (cc) groups. Statistical analysis was done using Student's *t*-test. "ns", not significant. The middle line in each box represents the median. Box edges and whiskers represent the 25th/75th and 2.5th/97.5th percentiles, respectively.

Table S1. Cell number of ICM and TE in SCNT control, SCNT TSA, SCNT *Kdm4d*, SCNT *Kdm4d+5b*, SCNT *Kdm4d*+TSA, SCNT *Kdm4d+5b*+TSA, and NF groups. Related to Figure 1c, d.

Group		Cell number of ICM (Mean ± SD)	Cell number of TE (Mean ± SD)
SCNT	Control	8.67 ± 3.22^{A}	45.33 ± 2.08^{a}
	TSA	9.67 ± 2.08^{B}	45.67 ± 6.11^{b}
	Kdm4d	14.33 ± 1.53^{C}	33 ± 2.65^{c}
	Kdm4d+5b	16.00 ± 3.00^{D}	33.67 ± 6.51^{d}
	Kdm4d+TSA	13.67 ± 5.86^{E}	34.67 ± 5.77^{e}
	Kdm4d+5b+TSA	23.67 ± 6.03^{F}	36.33 ± 6.51^{f}
NF		19.67 ± 1.16^{G}	33.67 ± 2.52^{g}

Each group has three biological replicates. For A versus G, B versus G, a versus g, b versus g, $p<0.05$. For C versus G, D versus G, E versus G, F versus G, c versus g, d versus g, e versus g, f versus g, ns, not significant.

Table S2. Pre-implantation development of SCNT embryos. Related to Figure 1e.

	2-cell% (Mean ± SD)	4-cell% (Mean ± SD)	morula% (Mean ± SD)	blastocyst% (Mean ± SD)
Control	100	70.73 ± 2.72	32.47 ± 4.05	12.57 ± 1.97[A]
TSA	100	76.13 ± 10.89	56.87 ± 13.95	36.57 ± 8.04[B]
Kdm4d	100	100	91.53 ± 7.81	66.6 ±5.76[C]
Kdm4d+5b	100	100	90.57 ± 10.05	71.13 ± 7.68[D]
Kdm4d+TSA	100	96.3 ± 6.41	88.33 ± 12.58	73.33 ± 2.89[E]
Kdm4d+5b+ TSA	100	97.63 ± 2.15	94.07 ± 2.50	74.90 ± 1.35[F]

Each group has three biological replicates. For A versus B, $p<0.01$. For A versus F, $p<0.0001$. For B versus F, $p<0.01$. For C versus F, D versus F, E versus F, ns, not significant.

Table S3. Cell number of ICM and TE in 4N NF and 4N NT (cc) groups. Related to Figure 2d, e.

Group	Cell number of ICM (Mean ± SD)	Cell number of TE (Mean ± SD)
4N NF	21.33 ± 9.87^{A}	33.33 ± 10.02^{a}
4N NT (cc)	27.00 ± 6.56^{B}	40.00 ± 11.79^{b}

Each group has three biological replicates. For B versus A, b versus a, ns, not significant.

Table S4. Post-implantation development of SCNT and reconstructed SCNT embryos. Related to Figure 5c, f, Figure S9b.

	No. of recipients	No. of blastocysts transferred	No. of implanted (% per ET) Mean ± SD	No. of Full-Term Pups (% per ET) Mean ± SD	Weight of pup at birth Mean ± SD (g)	Weight of placenta at birth Mean ± SD (g)
NT (sc)	5	57	27 (49.36 ± 27.70)^{A}	5 (8.9 ± 1.39)^{E}	1.37 ± 0.16^{I}	0.29 ± 0.10^{M}
4N NT (sc)	6	39	36 (92.75 ± 8.05)^{B}	12 (30.17 ± 16.80)^{F}	1.42 ± 0.20^{J}	0.16 ± 0.05^{N}
NT (cc)	5	69	35 (53.60 ± 14.58)^{C}	5 (8.18 ± 3.58)^{G}	1.43 ± 0.46^{K}	0.22 ± 0.05^{O}
4N NT (cc)	7	54	46 (85.20 ± 11.88)^{D}	16 (29.67 ± 5.07)^{H}	1.45 ± 0.16^{L}	0.14 ± 0.03^{P}

Number of recipients represent replicates. For B versus A, D versus C, $p<0.01$

For F versus E, $p<0.05$. For H versus G, $p<0.0001$.

For J versus I, L versus K, ns, not significant.

For N versus M, $p<0.01$, For P versus O, $p<0.001$
